# Cell-Driven Encapsulation of Chlorophyllin-Based Carbon Dots within Exosomes for Enhanced Photodynamic Therapy: miRNA Profiling Reveals Mechanistic Insights

**DOI:** 10.1021/acsami.5c18555

**Published:** 2025-12-01

**Authors:** Omur Besbinar, Recep Uyar, Emel Kirbas Cilingir, Ana Martín-Pardillos, Jose L. Hueso, Ahmet Ceylan, Ozge Ozgenc, Okan Ekim, Mehmet Altay Unal, Fikret Ari, Roger M. Leblanc, Jesus Santamaria, Acelya Yilmazer

**Affiliations:** † Stem Cell Institute, 37504 Ankara University, 06520 Ankara, Turkey; ‡ Department of Chemistry, 5452 University of Miami, 1301 Memorial Drive, Coral Gables, Florida 33146, United States; § 82976 Instituto de Nanociencia y Materiales de Aragon (INMA), CSIC-Universidad de Zaragoza, Campus Rio Ebro, Edificio I+D, C/Poeta Mariano Esquillor, s/n, 50018 Zaragoza, Spain; ∥ Department of Chemical Engineering and Environmental Technology (IQTMA), 16765 University of Zaragoza, 50018 Zaragoza, Spain; ⊥ Networking Research Center in Biomaterials, Bioengineering and Nanomedicine (CIBER-BBN), Instituto de Salud Carlos III, 28029 Madrid, Spain; # Instituto de Investigación Sanitaria (IIS) de Aragón, Avenida San Juan Bosco, 13, 50009 Zaragoza, Spain; ∇ Escuela Politécnica Superior, Universidad de Zaragoza, Crta. de Cuarte s/n, 22071 Huesca, Spain; ○ Department of Biomedical Engineering, Faculty of Engineering, Ankara University, 06830 Ankara, Turkey; ◆ The Graduate School of Health Sciences of Ankara University, 06110 Ankara, Turkey; ¶ Department of Histology Embryology, Faculty of Veterinary Medicine, Ankara University, 06110 Ankara, Turkey; & Department of Anatomy, Faculty of Veterinary Medicine, Ankara University, 06110 Ankara, Turkey; ● Department of Electrical Electronic Engineering, Faculty of Engineering, 06830 Ankara, Turkey

**Keywords:** exosomes, carbon dots, photodynamic therapy, miRNA profiling, drug delivery

## Abstract

Exosomes, nanoscale extracellular vesicles, have emerged as promising carriers in drug delivery due to their ability to bypass biological barriers, low toxicity, high stability, and intrinsic targeting capabilities. Mesenchymal stem-cell-derived exosomes (EXO_MSC_), with their natural tropism toward the tumor microenvironment, offer an ideal platform for enhancing therapeutic cargo delivery. In this study, we demonstrate an approach where red-emission chlorophyll-based carbon dots (Chl-CDs) were encapsulated within EXO_MSC_ through a cell-driven uptake mechanism, creating CD@EXO_MSC_. These exosomes achieved superior photodynamic therapy (PDT) efficacy, requiring 40 times less nanomaterial compared to freestanding Chl-CDs. Mechanistic insights from glioblastoma cell miRNA profiling revealed that the enhanced efficacy was mediated by the regulation of efflux transporter genes, oxidative stress responses, and endocytosis pathways. This work highlights the synergistic potential of combining photosensitizers and miRNA-rich exosomes to achieve targeted and sustained therapeutic delivery, paving the way for a multifaceted approach in cancer therapy.

## Graphical abstract

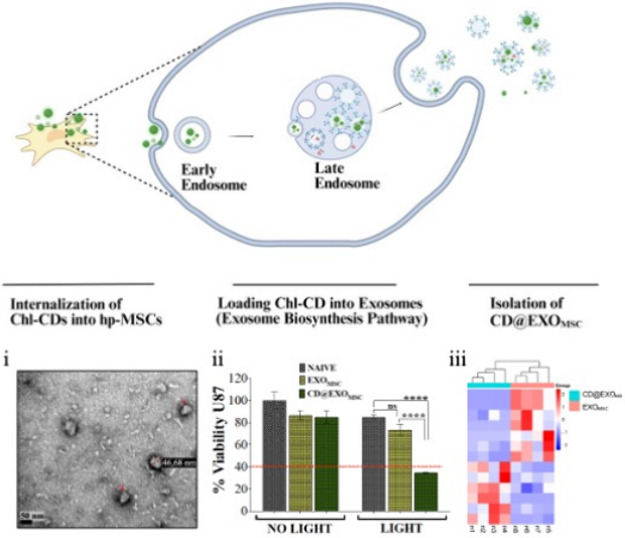

## 1 Introduction

Exosomes are nanoscale, double-lipid-layer extracellular vesicles secreted by cells that carry intrinsic biomolecules from their parental cells. Their unique properties have led to a rapidly expanding field of research in diagnostics, therapy, and drug delivery systems.^1^ Exosomes are valuable carriers of bioactive molecules, because of their ability to evade immune responses, cross biological barriers, and be modified to enhance targeting specificity.^2^ Their accumulation in tumor microenvironment (TME) and their efficient delivery of therapeutic agents into the recipient cell cytoplasm play key roles in increasing treatment efficacy. For instance, Lara et al.^3^ have demonstrated that exosomes accumulate in the TME at levels five times higher than achieved with free drug delivery systems. Similarly, Kim et al.^4^ have observed a 30-fold increase in cellular internalization with drug delivery via exosomes. One of the greatest challenges in cancer treatment is the development of multidrug resistance (MDR) in cancer cells.^5^ These extracellular vesicles might help overcome MDR, thus potentially explaining their enhanced efficacy in drug delivery. For example, Zhao et al.^6^ have reported that tetramethylpyrazine-loaded exosomes decrease the expression of key efflux proteins, such as P-gp and ABCC1, by approximately 70%. Extracellular vesicles have been employed to deliver therapeutic nanoparticles to tumors, achieving promising results.^7–9^ An important consideration in this context is ensuring that the materials are loaded while maintaining membrane integrity of vesicles; otherwise, the targeting capabilities of the vesicles could be hampered.

Carbon dots (CDs) are ultrasmall nanoparticles based on carbon, with remarkable physicochemical properties and biocompatibility, making them highly advantageous for biomedical applications.^10^ CDs can be easily synthesized from abundant natural sources, are colloidally stable in biological media and biosafe, which makes them ideal for use in near-infrared (NIR) imaging, diagnostics, and therapy via active generation of reactive oxygen species (ROS).^11–13^ Combining CDs with exosomal carriers is expected to increase therapeutic efficacy by decreasing the nanoparticles (NPs) degradation in the plasma, increasing target localization and cellular internalization, and controlling biodistribution, thereby minimizing adverse effects.^14^ For instance, Tiwari et al.^15^ have shown that exosomes carrying dacarbazine-conjugated carbon quantum dots (CQDs) achieve 4.5 times greater accumulation in tumor tissue and consequently enhance chemotherapeutic efficacy. Similarly, boron-containing carbon dots (BCDs) have been demonstrated to cross the blood brain barrier and target glioblastoma tumors effectively when they loaded into the exosomes, and therefore might have potential in neutron capture therapy.^16^

In cancer therapy, exosomes have been shown to play critical roles in the transport of miRNAs, which mediate molecular changes within target cells. Rather than merely profiling miRNAs, this approach emphasizes the functional implications of exosome-mediated miRNA delivery in modulating key regulatory pathways such as apoptosis, stress response, and immune modulation.^17,18^ Elucidating these molecular interactions might aid in understanding how treatments induce cellular responses and pave the way to more targeted and effective therapeutic strategies. This perspective aligns with this study’s aim of investigating the mechanistic effects of exosomal miRNA transfer on cancer cell dynamics. miRNAs loaded into exosomes or affected by exosome-mediated delivery have been shown to modulate gene expression networks and affect cellular pathways, including those associated with drug resistance and tumor progression.^19^ Specifically, miRNA transcriptome analysis can identify miRNAs that contribute to exosome-mediated photodynamic therapy (PDT) efficacy by targeting pathways associated with oxidative stress and efflux transporter regulation; this method therefore provides valuable insight into how exosome-loaded nanomaterials influence cellular responses to therapy.^20^

In our previous study, we synthesized chlorophyllin-based carbon dots (Chl-CDs) and explored their therapeutic potential.^21^ In cancer cells that internalize these red-emitting Chl-CDs, cell death is induced by ROS generation after excitation with LED at 520 nm. Molecular analyses have revealed that ferroptosis accompanies apoptosis during PDT treatment and enhances the therapeutic effect of Chl-CDs. This study aims at encapsulating Chl-CDs within exosomes derived from human placental mesenchymal stem cells (EXO_MSC_) through a cell-driven encapsulation approach to enhance their delivery efficiency and therapeutic potential in PDT. Exosomes from stem cells are especially interesting candidates in cancer therapy, as they have shown tropism toward tumors.^22,23^ Our underlying hypothesis was that exosome-encapsulated Chl-CDs (CD@EXO_MSC_) would be effectively internalized by cancer cells and would subsequently improve treatment outcomes by enabling targeted and sustained delivery of therapeutic agents. In vitro and in vivo analyses demonstrated that CD@EXO_MSC_ significantly enhanced cellular uptake and achieved superior therapeutic efficacy to freestanding Chl-CDs. Additionally, miRNA transcriptome analyses were conducted to elucidate the molecular mechanisms underpinning the exosomes’ enhanced therapeutic effects, with a focus on the regulatory roles of miRNAs in apoptosis, oxidative stress, and immune modulation pathways. The ability of the Chl-CDs photosensitizers to reach their targets via exosome-mediated delivery, together with the miRNA-rich composition of exosomes, enabled a multifaceted approach to enhancing the efficacy of PDT. This integrated strategy enables a comprehensive understanding of the interplay between exosome-mediated delivery and miRNA-driven molecular alterations in cancer therapy and offers new insights into the potential synergistic effects of these mechanisms.

## 2 Results

### 2.1 Upon the Internalization of Chl-CDs, hpMSCs Secrete Them into the Extracellular Environment Incorporated into Exosomes – a Cell-Driven Appraoch

The characterization and cytotoxicity analyses of Chl-CDs used here were reported in our previous study.^21^ Based on optical assessments, Chl-CDs exhibit a characteristic absorbance at 400 nm and emits red fluorescence at 650 nm (Figure S1A). TEM analysis revealed that the average particle size of the nanomaterial ranges between 2–3 nm (Figure S1B). The presence of copper in the composition was confirmed via MP-AES measurements (Figure S1C). The effects of Chl-CDs on cell viability in different cell lines are presented in the supplementary data (Figure S1D).

Chl-CDs were intended to be intrinsically loaded into exosomes produced by hpMSCs through the exosome biogenesis pathway. Chl-CDs internalization was evaluated using two fundamentally distinct techniques. ICP-MS analysis leveraged the detectable copper content of Chl-CDs to quantify cellular uptake (Figure 1A, left panel), while flow cytometry relied on their intrinsic fluorescence to monitor internalization (Figure 1A, right panel). The use of both elemental analysis and optical properties for tracking demonstrates the versatile nature of Chl-CDs. Notably, both methods consistently indicated that uptake peaked at the fourth hour and subsequently plateaued, thereby validating each other’s findings. Furthermore, the intrinsic fluorescence of Chl-CDs permitted sensitive detection of cellular uptake by flow cytometry, even at low doses (Figure S2A).

**Figure 1:**
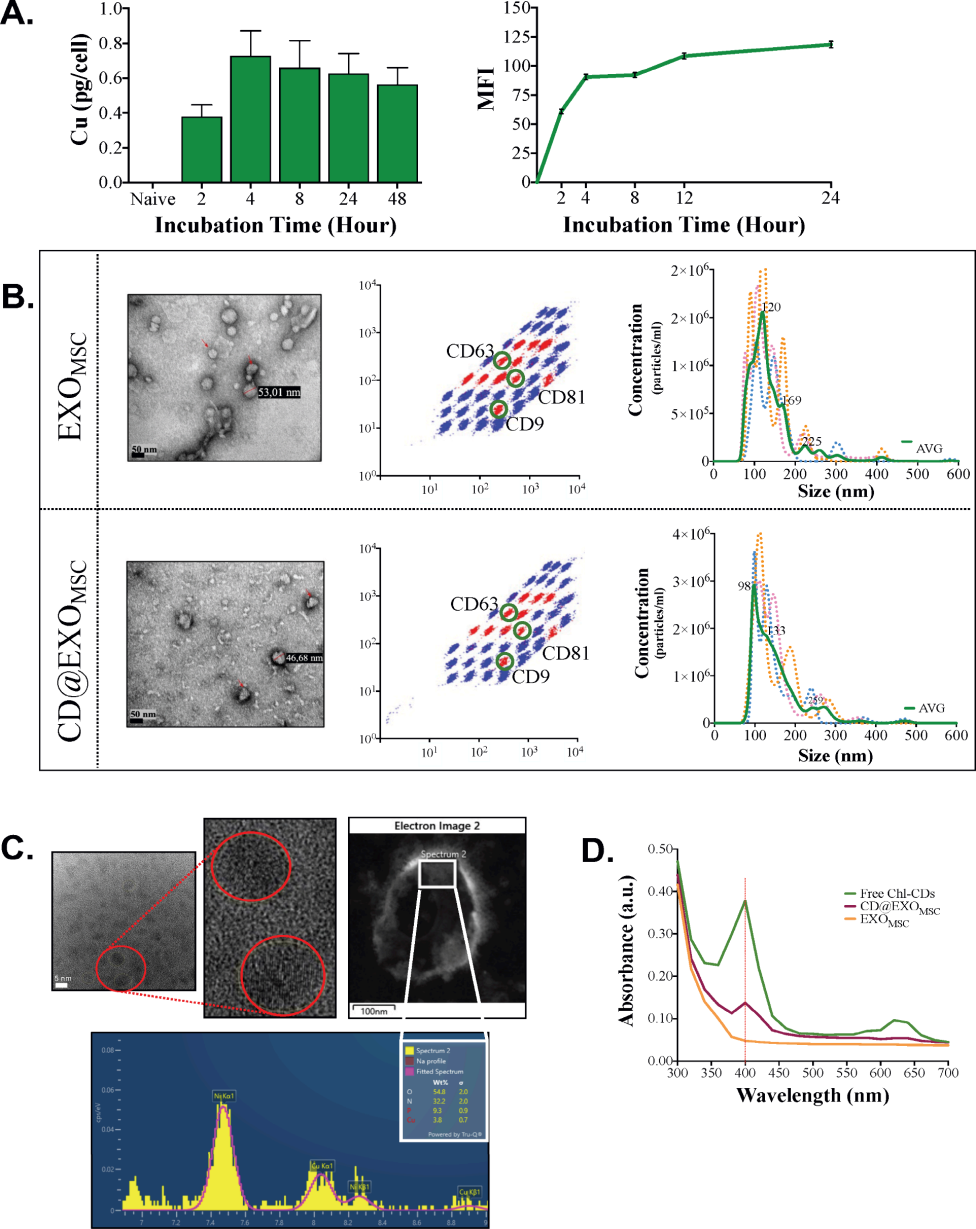
Characterization of CD@EXO_MSC_. (A) Chl-CD internalization: 300,000 cells/well hpMSC were incubated with 250 μg/mL Chl-CDs for various times. At the end of incubation, cells were counted, and cell pellets were analyzed with ICP-MS for Chl-CDs-originated copper element analysis (left panel). For confirmation of Chl-CDs internalization according to the intrinsic fluorescence properties of Chl-CDs, Chl-CD-treated hpMSCs were measured directly with flow cytometry at the end of the incubation periods (right panel) (MFI: mean fluorescence intensity). (B) Exosome characterization: the morphology of exosomes isolated from Chl-CDs-treated hpMSC culture supernatant was visualized with TEM (left panel). A MACPlex Exosome Kit was used to evaluate the expression of the exosome surface markers CD63, CD9, and CD81 by flow cytometry (center panel). Exosome size distribution and particle concentration were analyzed with NTA (right panel). (C) Cu detection: the detection of Chl-CDs in exosomes was evaluated with STEM-EDX analysis and HR-TEM, on the basis of the presence of the Cu element and the crystalline structure of free Chl-CDs, respectively. (D) Loading efficiency: a calibration curve was created with the absorbance values of free-Chl-CDs serial dilutions at 400 nm. The presence of the peak at 400 nm for Chl-CD-loaded exosomes (CD@EXO_MSC_), empty exosomes (EXO_MSC_), and free-Chl-CDs was demonstrated. The amount of Chl-CDs in the exosomes was calculated using the calibration curve of freestanding Chl-CDs.

The incorporation of Chl-CDs into the exosome biogenesis resulted in their release as Chl-CD-loaded exosomes (CD@EXO_MSC_). Control exosomes (EXO_MSC_) derived from hpMSCs not treated with Chl-CDs, as well as CD@EXO_MSC_ isolated from the hpMSC culture supernatant, were additionally characterized for the sake of comparison. On the basis of TEM imaging, both groups of exosomes exhibited spheroidal morphology. The average sizes of EXO_MSC_ and CD@EXO_MSC_ were 53.01 and 46.68 nm, respectively. Flow cytometry analysis confirmed the expression of the exosome-specific biomarkers CD9, CD63, and CD81. According to NTA measurements, the hydrodynamic sizes of CD@EXO_MSC_ and EXO_MSC_ were 152.5 ± 1.1 nm and 141.4 ± 2.8 nm, respectively (Figure 1B). The incubation of hpMSCs with Chl-CDs therefore did not significantly affect the characteristics of the exosomes. To confirm the incorporation of Chl-CDs into the exosomes, HAADF-STEM imaging, EDX analysis and HR-TEM analyses were performed. The crystalline structure and copper signals of Chl-CDs were detected in the CD@EXO_MSC_ (Figure 1C). Additionally, the specific peak recorded at 400 nm in CD@EXO_MSC_ was attributed to the presence of Chl-CDs (Figure 1D). Together, the crystalline structure of Chl-CDs, the presence of copper, and the specific absorbance value helped to validate the loading of Chl-CDs into CD@EXO_MSC_. The amount of Chl-CDs transported by the exosomes was calculated using the calibration curve generated from the absorbance of free Chl-CDs (Figure S2B). The CD@EXO_MSC_ contained approximately 0.262 ± 0.028 μg Chl-CDs per μg of exosomal protein. Finally, the stability of exosomes has been studied in FBS and analyzed by NTA. According to Figure S2C, the size of exosomes (CD@EXO_MSC_) increased slowly from 150.3 to 153.0 nm, and there was no statistical significance between different time points.

### 2.2 Exosome-Mediated Delivery of Chl-CDs to Cancer Cells Drives an Efficient PDT Response In Vitro

Cytotoxicity analysis was performed to determine the working dose and evaluate the effects of exosomes on cell viability. In all three cancer cell lines (U87-MG, U251-MG, and A549), exosomes at a concentration of 10 μg/mL decreased the cell viability to below 80%. Based on these findings, 5 μg/mL was established as the subcytotoxic working dose for exosomes (Figure 2A). Exosome uptake by cancer cells was evaluated with flow cytometry, on the basis of the intrinsic red fluorescence of Chl-CDs. All three cancer cell types demonstrated internalization in more than 50% of the cells. Therefore, the optical properties of Chl-CDs are suitable for use in tracking, without a need for additional fluorochrome conjugation. Flow cytometry analysis detected the presence of Chl-CDs even at low doses in the exosomes (Figure 2B). In vitro evaluation of exosome-mediated PDT treatment revealed that all three cancer cell lines responded positively to the therapy. Light exposure after CD@EXO_MSC_ incubation led to significant cell death in the cancer cells. Cell viability decreased to as low as approximately 35, 50, and 30% in U87-MG, U251-MG, and A549 cells, respectively. Therefore, the glioblastoma-derived U87-MG and U251-MG cell lines were more sensitive to the therapy than the A549 lung cancer cell line, because EXO_MSC_ without Chl-CDs also induced cell death in A549 cells after light irradiation (Figure 2C). The in vitro PDT dose with exosomes (5 μg/mL), corresponding to approximately 1.31 μg/mL Chl-CDs, required 40-fold lower amounts of Chl-CDs than required in PDT with free Chl-CDs to achieve a similar cell viability of approximately 40%, as observed in our previous study.^21^

**Figure 2:**
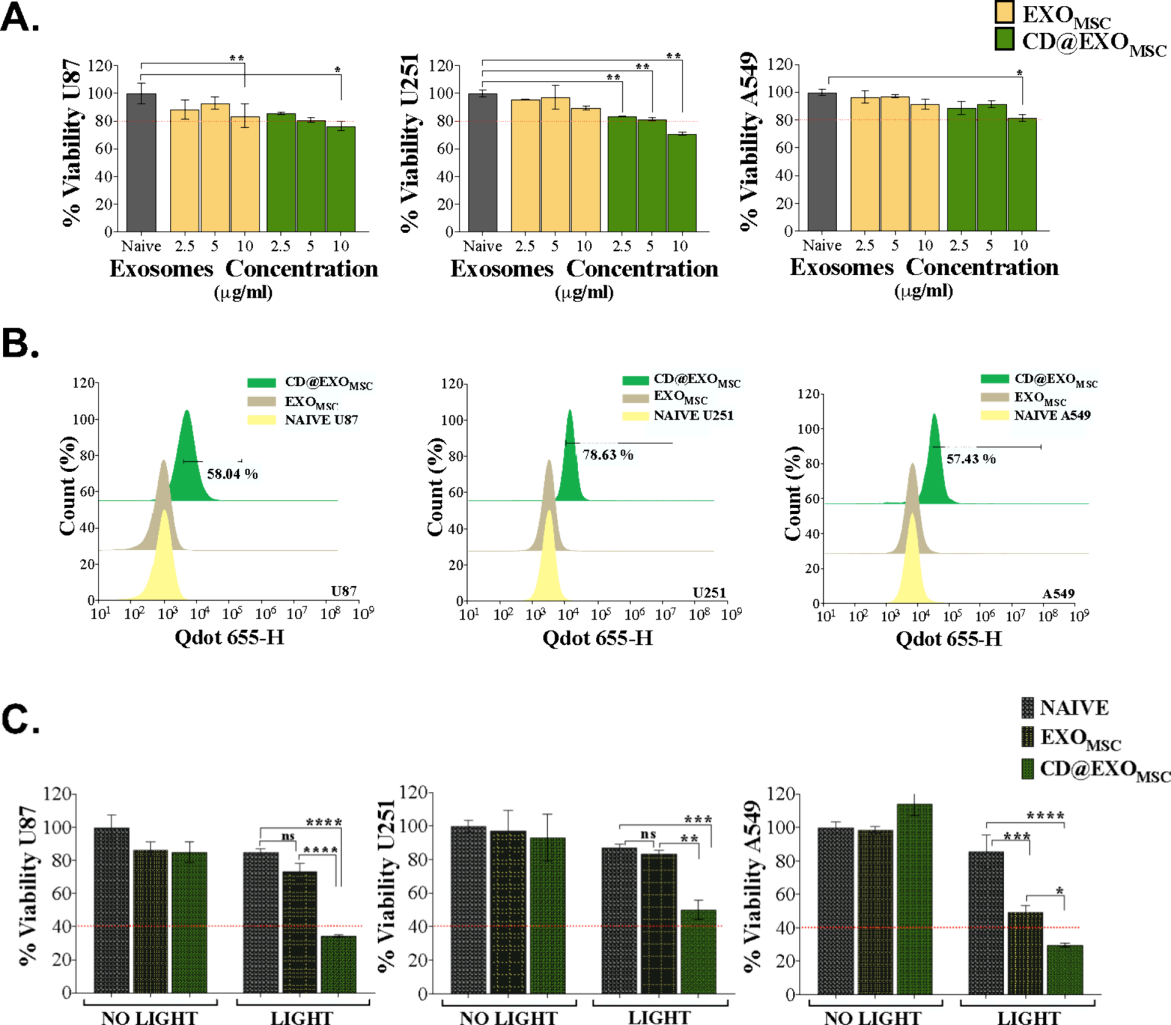
Exosome-mediated PDT. (A) Exosome toxicity: exosomes at three concentrations were applied to U87-MG, U251-MG, and A549 cancer cells for 4 h. Exosome cytotoxicity was evaluated with LDH assays. (B) Exosome internalization: U87-MG, U251-MG, and A549 cancer cell lines were treated with 5 μg/mL exosomes for 4 h, and the cells were directly analyzed with flow cytometry. The internalization of exosomes into cancer cells was assessed without any additional staining, on the basis of the intrinsic fluorescence of Chl-CDs in the exosomes. (C) Exosome-mediated PDT: U87-MG, U251-MG, and A549 cancer cells were treated with 5 μg/mL exosomes for 4 h. After incubation, the cells were exposed to 520 nm LED light for 1 h. Cell viability was assessed with LDH assays. (The groups were evaluated with respect to the control group with two-way ANOVA followed by Tukey’s multiple comparison test. Statistical significance was indicated as follows: (ns), 0.1234; *, *p* < 0.0332; **, *p* < 0.0021; ***, *p* < 0.0002; ****, *p* < 0.0001.)

### 2.3 Molecular Mechanisms Underlying Cell Death Are More Effectively Orchestrated in Exosome-Mediated PDT Compared to Free Chl-CDs-Mediated PDT

To better elucidate the molecular mechanisms underlying the therapeutic effects, miRNA sequencing analysis was conducted to compare the groups treated with PDT, CD@EXO_MSC_+LIGHT or free Chl-CDs+LIGHT, in U87-MG glioblastoma cells. This comparative analysis specifically focused on identifying differentially expressed miRNAs and their associated regulatory pathways. The study revealed significant alterations in biological pathways associated with apoptosis, oxidative stress, and immune modulation, driven by miRNA-mediated mechanisms. The results were systematically visualized through detailed plots, which offered insights into the distinct molecular effects of exosome-mediated versus free Chl-CDs delivery in the context of PDT. After a comprehensive analysis of miRNA expression patterns, it was observed distinct transcriptional profiles between groups. Principal component analysis (PCA) demonstrated a pronounced separation between treatment regimens (Figure 3A), thus suggesting that CD@EXO_MSC_+LIGHT elicited a unique transcriptional response from the other treatments. Further insights into miRNA regulation were provided by a heatmap (Figure 3B), which indicated significant differences in miRNA expression levels across treatment groups. The clustering patterns indicated that exosome-mediated delivery substantially impacted miRNA expression, and distinct profiles emerged between treated groups. In addition, differential expression analysis, visualized by a volcano plot (Figure 3C), identified key upregulated and downregulated miRNAs in response to CD@EXO_MSC_+LIGHT. Among these, *hsa-miR-27a-5p*, and *hsa-miR-6741–5p* were notably upregulated, whereas *hsa-miR-45*08 and *hsa-miR-4449* were downregulated. These miRNAs might be critical in mediating responses to oxidative stress, oncogenic pathways, and tumor suppression, thus indicating complex regulatory mechanisms activated by the CD@EXO_MSC_+LIGHT treatment.^24–28^ To further explore the functional implications of these miRNA alterations, pathway enrichment analysis (Figure 3D), emphasizing key biological processes affected by CD@EXO_MSC_+LIGHT. This analysis highlighted the activation of tumor suppressor pathways, such as “oncogene induced senescence” and “transcriptional regulation by TP53,” as well as stress-response pathways, including “HSF1-mediated heat shock response” and “oxidative stress induced senescence.” These findings suggested a central role of heat shock transcription factor 1 (HSF1) in managing cellular stress and maintaining tumor suppression in response to the oxidative damage triggered by PDT. The complexity of miRNA-pathway interactions was further illustrated by mapping miRNAs to their associated pathways with a chord plot (Figure 3E, detailed miRNA–gene associations underlying these pathways are provided in Tables S1 and S2). This visualization uncovered an intricate network of miRNAs involved in oncogenesis, the oxidative stress response, and tumor suppression, thereby underscoring the multifaceted regulatory landscape modulated by CD@EXO_MSC_+LIGHT. By identifying specific miRNAs driving these pathways, this analysis offered valuable insights into potential therapeutic targets to enhance treatment efficacy. Collectively, these findings revealed the molecular and cellular changes induced by Chl-CD-loaded exosomes in combination with PDT and emphasized the miRNA-mediated pathways governing tumor progression and suppression. The miRNA pathway enrichment analysis demonstrated the multifaceted regulatory effects induced by CD@EXO_MSC_+LIGHT and uncovered intricate networks involved in tumor suppression, the oxidative stress response, and cellular adaptation. Building upon these insights, further attention was directed toward understanding the role of carbon dot incorporation in shaping exosomal miRNA profiles.

**Figure 3:**
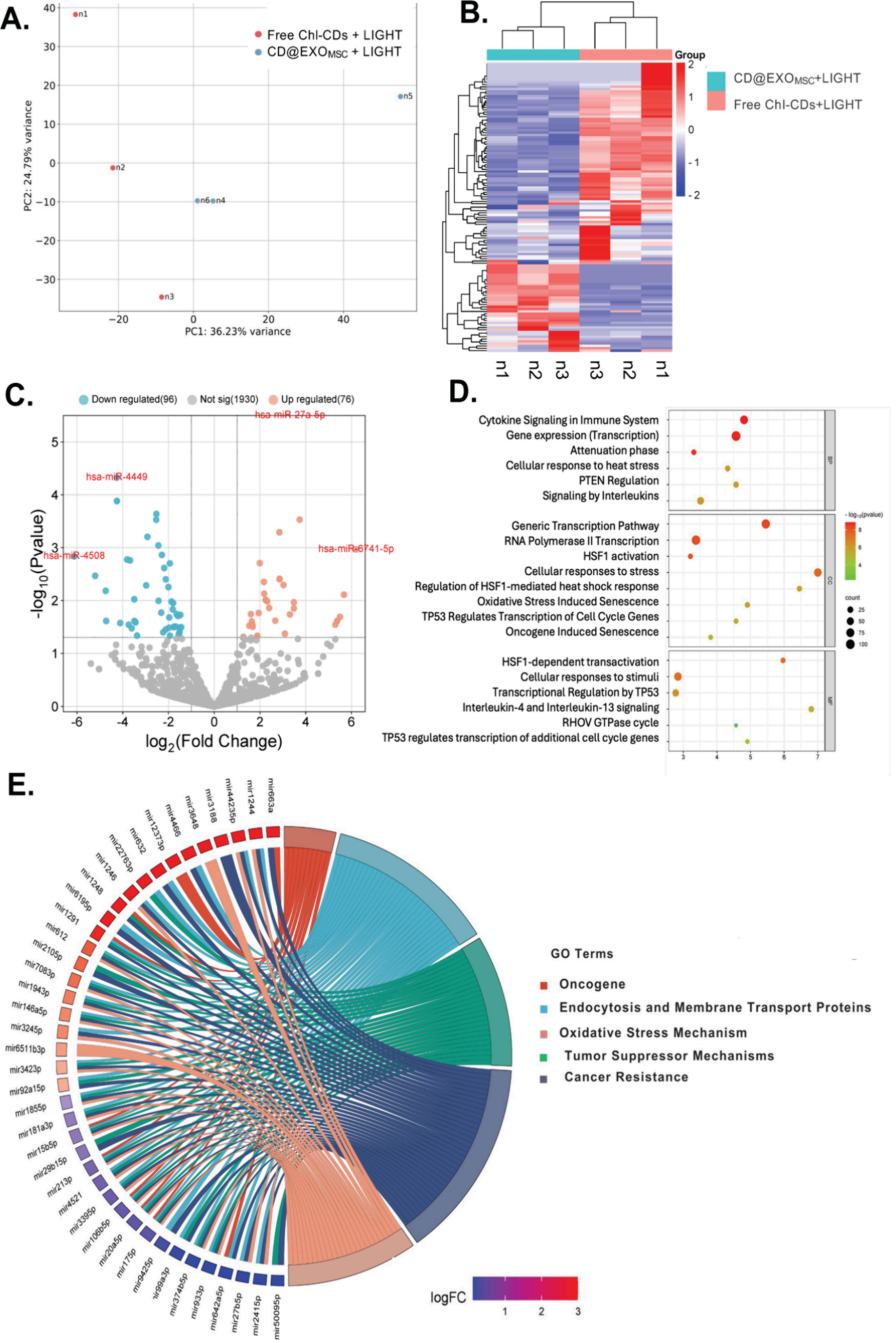
Analysis of miRNA expression and pathway enrichment between the CD@EXO_MSC_+LIGHT and free ChI-CDs+LIGHT groups. (A) PCA plot showing the separation between CD@EXO_MSC_+LIGHT versus free ChI-CDs+LIGHT groups, on the basis of miRNA expression profiles. (B) Heatmap of significantly differentially expressed miRNAs between groups. (C) Volcano plot of differentially expressed miRNAs, highlighting upregulated (red) and downregulated (blue) miRNAs. (D) With the results of GO analysis, a pathway enrichment plot was generated, showing significantly enriched pathways and their associated miRNAs. (E) Chord plot illustrating the relationship between differentially expressed miRNAs and their target pathways, categorized into oncogenesis, oxidative stress mechanisms, endocytosis and membrane transport, tumor suppressor mechanisms, and cancer resistance.

### 2.4 Chl-CDs Treatment Alters the miRNA Profile of hpMSC-Derived Exosomes and Contributes to the Therapeutic Response in Recipient Cells

Subsequent analysis revealed distinct transcriptional changes between the CD@EXO_MSC_ and EXO_MSC_ groups, and consequently shed light on the specific modifications imparted by Chl-CDs. The observed divergence in miRNA expression profiles underscored the transformative effect of CD loading on exosomal functionality; these findings might pave the way to deeper exploration of their therapeutic potential. Comparative miRNA analysis between Chl-CD-loaded exosomes (CD@EXO_MSC_) and empty exosomes (EXO_MSC_) further elucidated the role of Chl-CDs in amplifying cellular responses in hpMSC. Evaluation of miRNA expression patterns between the CD@EXO_MSC_ and EXO_MSC_ groups revealed distinct transcriptional profiles highlighting the effects of Chl-CDs loading on exosomal miRNA content. The clear separation between the CD@EXO_MSC_ and EXO_MSC_ groups in PCA indicated that Chl-CDs incorporation significantly modified the miRNA profiles of the exosomes (Figure 4A). Further assessment of miRNA expression with a heatmap revealed differentially expressed miRNAs that formed distinct clusters distinguishing the CD@EXO_MSC_ group from the empty exosome group. These clustering patterns highlighted miRNAs potentially involved in stress responses, cellular adaptation, and regulatory processes after Chl-CDs-loaded exosome uptake (Figure 4B). Additionally, a volcano plot highlighted specific upregulated miRNAs, such as hsa-miR-431–3p and hsa-miR-483–3p, alongside downregulated miRNAs, such as hsa-miR-648e-3p and hsa-let-7e-3p (Figure 4C). Pathway enrichment analysis identified processes associated with cellular stress and gene regulation, such as “HSF1 activation,” “HSF1-mediated heat shock response,” and PTEN-related pathways, which were also affected in PDT-treated groups. This analysis suggested that Chl-CDs loaded in exosomes enhanced the activation of oxidative stress pathways and miRNA-mediated regulatory mechanisms, and therefore might potentially increase cellular sensitivity to oxidative stress (Figure 4D). The implication of miRNAs in cell cycle regulation and stress-response pathways reinforces the distinctions observed in the previously described free Chl-CDs + Light-treated groups (Figure S3A). Compared with the effects of free Chl-CDs+LIGHT, exosome-encapsulated CD@EXO_MSC_+LIGHT demonstrated a distinct and more pronounced modulation of key signaling pathways previously observed in the PDT-treated groups. Notably, these pathways, including oxidative stress and gene regulation mechanisms, were consistently enriched in the free Chl-CDs+LIGHT group with respect to the control. However, further enrichment and enhanced activation of these pathways in the CD@EXO_MSC_+LIGHT group with respect to the free Chl-CDs+LIGHT group suggested that the exosomes not only facilitated more efficient delivery of Chl-CDs but also amplified their effects on cellular processes. This amplification might stem from the synergistic contributions of exosomal miRNAs, which are likely to augment the therapeutic effects by driving more robust engagement of these regulatory pathways. Further miRNA-gene network analysis, conducted to compare the CD@EXO_MSC_+LIGHT and free Chl-CDs+LIGHT groups, revealed interactions between differentially expressed miRNAs and target genes. TP53 emerged as a central regulatory hub, along with other critical genes such as MDM2, CSNK2A1, and YY1. This network suggested that miRNAs such as hsa-miR-150-5p and hsa-miR-21-3p might modulate TP53 and related pathways, and possibly affect stress responses and DNA damage responses induced by PDT, in agreement with previously observed HSF1-mediated stress mechanisms (Figure S3B). Collectively, these findings indicated that Chl-CDs within exosomes might prime cells for a heightened oxidative stress response and potentially enhance the efficacy of subsequent PDT treatments. The consistent activation of stress-response pathways across these datasets underscored the therapeutic potential of combining Chl-CDs-loaded exosomes with PDT to develop targeted cancer therapies.

**Figure 4:**
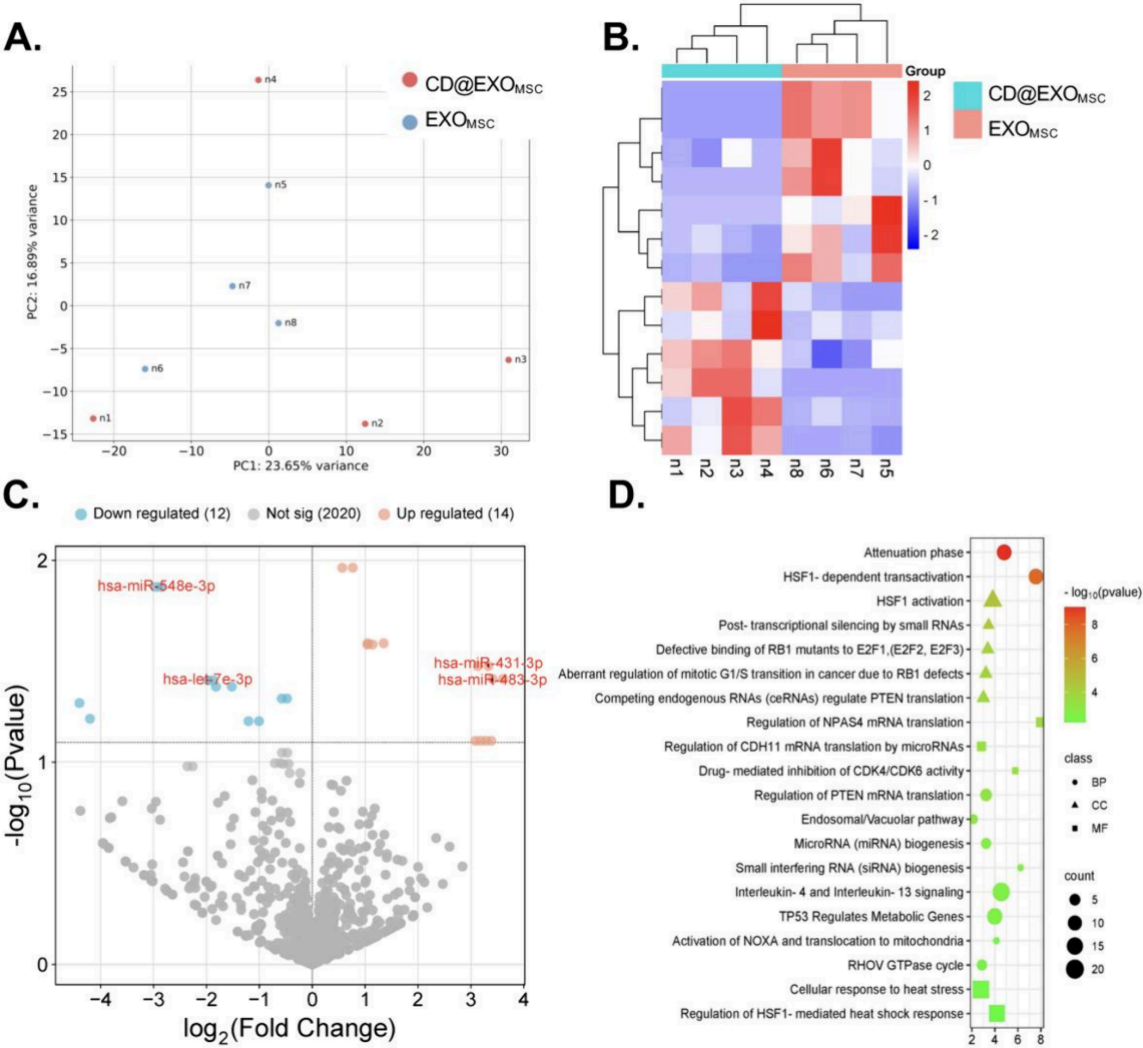
Analysis of miRNA expression and pathway enrichment between the CD@EXO_MSC_ and EXO_MSC_ groups. (A) PCA plot showing the separation between CD@EXO_MSC_ and EXO_MSC_ groups, on the basis of miRNA expression profiles. (B) Heatmap of significantly differentially expressed miRNAs between groups. (C) Volcano plot of differentially expressed miRNAs, highlighting upregulated (red) and downregulated (blue) miRNAs. (D). With the results of GO analysis, a pathway enrichment plot was generated, indicating significantly enriched pathways and their associated miRNA.

### 2.5 Chl-CDs-Loaded Exosomes Accumulate in Tumor Tissue and Elicit Significant Tumor Regression After PDT

After confirming the efficacy of exosome-mediated photodynamic therapy (PDT) in vitro, we proceeded to in vivo studies. For this, U87-MG glioblastoma cells were subcutaneously injected into the right dorsal region of Balb/C nude mice to establish a tumor model. The animals were then randomly assigned into five groups: naive, EXO_MSC_, CD@EXO_MSC_, EXO_MSC_+LIGHT, and CD@EXO_MSC_+LIGHT. The study flow is illustrated in Figure 5A. In the group treated with the combination of CD@EXO_MSC_ and LIGHT, compared with the naive group, approximately 50% tumor regression was observed (Figure 5B). In our previous study, free Chl-CDs+LIGHT treatment was performed in three cycles.^21^ However, in the exosome-mediated PDT, only a single dose of 40 μg exosomes was administered intravenously. This corresponds to approximately 10.48 μg of Chl-CD, which according to the literature is an extremely low dose for intravenous applications.^29,30^ This result might be attributable to the accumulation of exosomes in the tumor tissue and consequently the higher uptake of Chl-CDs by tumor cells. Exosome biodistribution was evaluated based on the intrinsic fluorescence emission of Chl-CDs carried by the exosomes, which showed extensive accumulation in the tumor tissue (Figures 5C and S4A). For biosafety assessments, the general health status and behavior of the animals were monitored over a two-week period. Compared to the group injected with PBS, no behavioral or physiological impairments were observed in the free Chl-CD and CD@EXO_MSC_ groups. In the randomly assigned mouse groups, no weight loss was detected during the 14-day period following intravenous administration, with body weights remaining in the range of 20–22 g (Figure 5D). Analysis of critical serum parameters revealed no deviations from established reference ranges (Figure 5E). Tumor tissue sections revealed preserved tumor morphology in the naive group. In rapidly growing tumors, early necrotic areas were apparent. In contrast, the CD@EXO_MSC_+LIGHT treatment group showed extensive cell death, marked tumor dissociation, and significant tumor shrinkage; these effects were more pronounced than those in the naive group. Moreover, this treatment group exhibited substantial disruption and fragmentation of tumor structure. In the other treatment groups, minor decreases in tumor size relative to that in the naive group were observed, although these changes were not histomorphologically prominent (Figure 5F). Examination of internal organs revealed no pathological findings, tissue integrity disruption, or material accumulation (Figure S4B). To assess the effects of different treatments on cell proliferation and apoptosis, immunohistochemical staining for PCNA and Caspase 3 was performed. PCNA expression was significantly reduced in the CD@EXO_MSC_+LIGHT group compared to the EXO_MSC_+ LIGHT, EXO_MSC_, and Naive groups (*p* ≤ 0.01), suggesting a marked decrease in proliferative activity following this treatment. Conversely, Caspase 3 expression was significantly elevated in the CD@EXO_MSC_+ LIGHT group relative to the EXO_MSC_+ LIGHT, EXO_MSC_, and Naive groups (*p* ≤ 0.01), indicating enhanced apoptotic activity. No statistically significant differences were observed among the remaining groups for either marker (*p* > 0.05) (Figure 5F).

**Figure 5:**
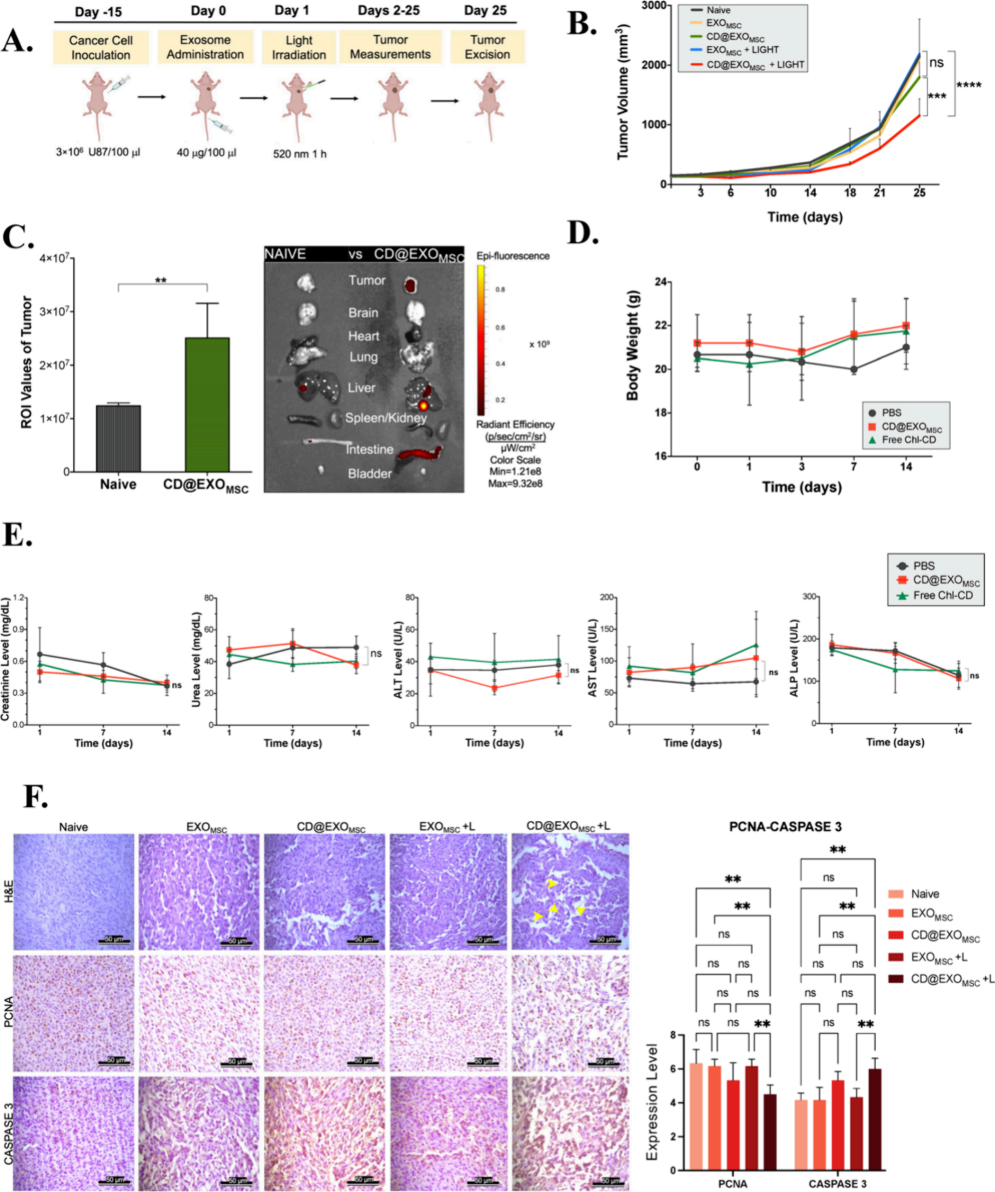
In vivo studies. (A) Study flow: for the orthotopic cancer model, 3 × 10^6^ U87-MG cells were injected into BALB/c nude mice. After the tumors reached an appropriate size, exosomes were injected, and irradiation with light was performed. (B) Tumor measurements: the mice were randomly divided into groups of five. The mice were intravenously administered 40 μg/mL of either empty exosomes (EXO_MSC_) or CD-loaded exosomes (CD@EXO_MSC_). Twenty-four h later, the tumor regions of the light treatment group were irradiated with 520 nm light for 1 h. Tumor volumes were measured every 3–4 days. (C) Biodistribution: 40 μg/mL exosomes (CD@EXO_MSC_) were injected intravenously into the mice. IVIS imaging was performed 24 h later. (D) Weight change: the body weights were monitored for 14 days following intravenous administration of 40 μg/mL exosomes (CD@EXO_MSC_) and 250 μg/mL free Chl-CD. (E) Biochemical assessment: critical serum biomarkers were evaluated following the administration, and the reference ranges were as follows: urea (17–71 mg/dL), creatinine (0.2–0.9 mg/dL), AST (54–298 U/L), ALT (17–77 U/L), and ALP (35–96 U/L). (F) Histology of tumors: formalin-fixed tumor sections were stained with hematoxylin and eosin (H&E). Immunohistochemical (IHC) staining was performed to assess apoptosis and proliferation using Caspase 3 and PCNA biomarkers, respectively. PCNA and Caspase 3 expression levels were analyzed separately using one-way ANOVA, followed by Tukey’s post hoc test for pairwise group comparisons. A p-value below 0.05 was considered statistically significant. Statistical significance levels are indicated as *p < 0.05, ** ≤ 0.01, *** ≤ 0.001, **** ≤ 0.0001.

## 3 Discussion

The tracking and detection of organic structures like Carbon Dots is more challenging than metal-based nanomaterials.^31^ Generally, CDs are hybridized with doped metals, fluorochromes, or drugs, which not only contribute to enhancing their therapeutic efficacy but also facilitate their detection through analytical techniques such as HPLC and ICP-MS.^32–34^ The chlorophyllin-based CDs used in this study provided ease of use without a need for external manipulation, owing to their red emission and the copper metal element contained within their core. In therapeutic applications, carrier systems, such as nanosize vesicles, may contribute to nanomaterial stability, enhance treatment safety, and increase therapeutic efficacy.^35^ Li et al.^16^ have investigated the effects of boron-containing Carbon Dots (BCDs) loaded-macrophage-derived exosomes in neutron capture therapy. In the study, BCDs were directly incubated with exosomes for loading. The contribution of exosomes in enhancing the therapeutic efficacy of BCDs was emphasized. Kazeminava et al.^34^ have demonstrated that methotrexate-conjugated carbon dots, loaded into breast cancer-derived exosomes with ultrasound assistance, enhanced their chemotherapeutic efficacy. In another study, Tiwari et al.^15^ have demonstrated that dacarbazine-primed carbon quantum dots were loaded into breast cancer cell-derived exosomes via sonication, resulting in enhanced chemotherapeutic efficacy. All these studies have demonstrated the enhanced therapeutic efficacy of CDs through the use of exosomes. Consistent with these findings, our results indicate that employing exosomes as carriers facilitates highly effective therapy while requiring significantly reduced amounts of Chl-CDs. Techniques frequently used in encapsulation processes to enhance loading capacity, such as sonication, electroporation, and external incubation with exosomes, can lead to unfavorable outcomes such as exosome membrane damage, aggregation, or material retention on the exosomal membrane surface.^36,37^ Maintaining adhesion molecules and membrane integrity on the exosome surface is critical for targeting the tumor microenvironment.^38,39^ In this study, we aimed to use the natural biogenesis pathway of exosomes by simply incubating Chl-CDs with hpMSCs and allowing exosome generation to take place. The results demonstrate that Chl-CDs were successfully loaded into exosomes by hpMSCs and that these CD-loaded extracellular vesicles maintained their targeting properties after loading. Thus, this study reports the first investigation of the efficacy of photodynamic therapy of this natural Chl-CDs encapsulation strategy, to our knowledge.

Exosomes are considered promising delivery vehicles, because of their nanoscale size, target-specific tropism, biocompatibility, and low-toxicity profiles. Additionally, their rich biomolecular content provided by their donor cells might contribute to therapeutic efficacy.^40^ This study demonstrated the integration of exosome technology with nanomaterial science, which achieved successful outcomes in both in vitro and in vivo photodynamic therapy applications. The mechanisms underlying the therapeutic enhancement facilitated by exosomes can be examined according to two main aspects. The first is the communication of exosomes with tumor cells. The exosomes were successfully delivered and substantially accumulated in tumor tissues, and subsequently augmented the transfer of exosomal content, including Chl-CDs, into cancer cells. Key features of exosomes, such as their nanoscale size, specific components on their membrane surfaces, ability to resist elimination within the body, and natural affinity for tumor tissues, are particularly important.^41^ Furthermore, exosome-mediated delivery might enhance cellular uptake relative to free Chl-CDs by engaging distinct uptake mechanisms, thus potentially facilitating lysosomal escape within cells, modulating cancer cell stress-response strategies, or establishing unique platforms for cell death pathways, owing to the biomolecular content carried within exosomes.^42^

The second supportive factor provided by exosomes is their natural content derived from donor cells. Exosomes, particularly through their miRNA cargo, can modulate cellular mechanisms in recipient cells and consequently enhance the efficacy of transferred therapeutic agents^43,44^ · Notably, incubation of hpMSCs with Chl-CDs has been observed to significantly alter exosomal miRNA profiles. Therefore, exosomes carrying Chl-CDs might transfer miRNAs with distinct profiles and lead to activation of various pathways in the context of PDT therapy. Additionally, in response to the increased cellular stress induced by light application, various types of cell death might be triggered. For instance, regulation of the expression of genes associated with cuproptosis—such as FDX1, LIAS, PDHB, and SLC31A1—has been observed. These genes are integral to the cuproptosis pathway, a form of regulated cell death induced by copper accumulation and mitochondrial stress.^45,46^ Ferredoxin 1 (FDX1) is a key regulator in this process, whereas lipoic acid synthetase (LIAS) and pyruvate dehydrogenase E1 beta subunit (PDHB) are involved in mitochondrial metabolism.^47^ Solute carrier family 31 member 1 (SLC31A1) functions as a high-affinity copper uptake protein facilitating copper transport into cells.^48^ The copper element within Chl-CD has been hypothesized to potentially trigger cuproptosis after delivery into cells.^49^ Copper is a metal element known to induce programmed cell death, such as ferroptosis, apoptosis, and cuproptosis, by catalyzing Fenton-like reactions and exhibiting reactive responses across a wide pH range. Furthermore, copper has been reported to deplete glutathione (GSH),^50^ which plays a role in scavenging ROS within tumor tissues^51^ and also to catalyze transamination within tumor cells, significantly depleting the pool of key molecules such as glutamine and alanine.^52^ Therefore, an increase in copper levels within the tumor microenvironment is believed to enhance the efficacy of ROS-based therapeutic approaches, such as PDT. The gene regulation suggests that exosome-mediated delivery of Chl-CDs might sensitize cells to cuproptosis and therefore enhance the therapeutic efficacy of PDT.

The miRNA transcriptome analysis conducted in this study provided critical insights into the cellular pathways and mechanisms underlying the enhanced efficacy of PDT with Chl-CDs-loaded exosomes as delivery vehicles. The distinct separation between the CD@EXO_MSC_+LIGHT and free Chl-CDs+LIGHT groups, as indicated by PCA and clustering patterns, reflected a unique miRNA signature in response to exosome-mediated PDT. This shift suggested that exosome encapsulation not only facilitates the cellular uptake of photosensitizers but also modulates cellular response pathways and might potentially enhance therapeutic effectiveness in ways not achievable by free nanomaterials alone. A notable finding among the differentially expressed miRNAs was the upregulation of *hsa-miR-27a-5p*, a previously identified tumor-suppressive miRNA. Studies have shown that *miR-27a-5p* is associated with diminished metastatic potential and improved patient outcomes in breast cancer, particularly in HER2-amplified tumors.^32^ Regulation of *miR-27a-5p* in CD@EXO_MSC_+LIGHT-treated cells suggested that this miRNA might contribute to enhanced therapeutic efficacy by modulating tumor-suppressive pathways and promoting apoptosis under oxidative stress conditions induced by PDT.^33^ *miR-27a* in many tumors supports tumor progression by reinforcing survival, growth, and invasiveness through activation of the PI3K/AKT and Wnt/β-catenin pathways. Because of its oncogenic properties, this miRNA might serve as a potential therapeutic target: inhibiting its expression might decrease tumor aggressiveness and improve treatment outcomes in patients with cancer.^27^ Other subtypes of hsa-*miR-27a* have also been associated with chemotherapy resistance in glioblastoma therapies.^26^ *miR-4449* has been identified as an oncogenic miRNA in multiple cancers, including gastric and colorectal cancer. In gastric cancer, *miR-4449* is regulated by circEIF4G3, a circular RNA that suppresses *miR-4449* activity, thereby inhibiting the SIK1 pathway and decreasing cancer cell proliferation and metastasis.^53^ In colorectal cancer, *miR-4449* promotes tumor progression by downregulating SOCS3, increasing STAT3 pathway activation, and consequently enhancing cell proliferation and inhibiting apoptosis.^25^ These studies highlight the role of *miR-4449* in tumor growth and suggest that it might serve as a potential target for therapeutic intervention in diverse cancers. Another study has demonstrated that *miR-6741–3p* plays a tumor-suppressive role in oral squamous cell carcinoma by directly targeting the oncogene SRSF3, and subsequently decreasing cell proliferation and tumor growth.^24^ These miRNAs are believed to play roles in modulating tumor suppressor pathways and stress adaptation mechanisms, thus highlighting their potential as regulatory mediators in PDT-induced cell death.

Our pathway enrichment analysis aligns with an emerging focus on tumor suppression mechanisms, notably through TP53-associated transcriptional regulation and the HSF1-mediated heat shock response pathway. These pathways play critical roles in modulating cellular resilience against oxidative damage induced by PDT. Specifically, HSF1 activation has been shown to orchestrate cellular defense mechanisms by stabilizing protein structures and decreasing proteotoxic stress, thereby counteracting the apoptosis and oxidative damage central to PDT’s efficacy.^54^ Moreover, the identified miRNAs—*hsa-mir-125a-3p*, *hsa-mir-3*0*a-3p*, *hsa-mir-629–5p*, *hsa-mir-548e-3p*, and *hsa-mir-483–3p—*might play crucial roles in regulating these stress response pathways. For instance, *hsa-mir-125a-3p* has been associated with the regulation of cellular oxidative stress and apoptosis, and it serves as a modulator in stress-responsive pathways and influencing tumor suppression mechanisms.^55,56^ Additionally, *hsa-mir-3*0*a-3p* ^57,58^ and *hsa-mir-483–3p* ^59^ have been implicated in apoptosis regulation and cellular stress responses, and therefore might enhance PDT efficacy by promoting cell cycle arrest and sensitizing tumor cells to oxidative stress. Collectively, these miRNAs are likely to contribute to the observed therapeutic effects of CD-loaded exosome treatment, in alignment with the enriched HSF1-associated stress response and TP53-regulated pathways. The activation of TP53 as a central node within the miRNA-gene interaction network underscores the potential role of p53-regulated pathways in the therapeutic response to PDT. TP53, along with other critical regulators such as MDM2 and CSNK2A1, is involved in a coordinated regulatory response to cellular stress that might potentially enhance the apoptotic and ferroptotic responses in cancer cells treated with CD@EXO_MSC_ and light. Previous studies have shown that miRNAs such as *hsa-miR-21–3p* ^60,61^ and *hsa-miR-15*0*-5p* ^62^ influence TP53 signaling, thus indicating a role of miRNAs in fine-tuning the oxidative stress and DNA damage response.

## 4 Conclusions

This study underscores the substantial potential of exosome-based delivery systems in advancing the therapeutic efficacy of PDT through the innovative use of Chl-CDs. By leveraging the intrinsic properties of mesenchymal stem cell-derived exosomes, including their biocompatibility, stability, tumor-specific tropism, and ability to carry bioactive molecules, the therapeutic landscape of PDT has been profoundly enriched. The cell-driven natural encapsulation of Chl-CDs within exosomes not only markedly enhanced the targeted delivery of photosensitizers to tumor cells but also achieved substantial therapeutic efficacy with substantially lower nanomaterial doses. This efficiency is attributed to exosomes’ superior cellular uptake capabilities and their roles in modulating miRNA profiles, which collectively amplify the cellular stress response and tumor suppression mechanisms.

This study not only advances understanding of exosome-mediated PDT but also lays groundwork for future research aimed at exploring the broader applicability of this strategy. Moreover, the development of exosome platforms capable of encapsulating a variety of nanomaterials or therapeutic agents could unlock new possibilities for precision oncology. Ultimately, the integration of exosome-based delivery systems into clinical practice has the potential to revolutionize cancer treatment, by offering a targeted, efficient, and minimally invasive approach to combating this complex disease.

This study highlights the therapeutic advantages of Chl-CDs-loaded exosomes in photodynamic therapy, emphasizing their ability to enhance miRNA regulation, activate stress-response pathways, and facilitate efficient cellular uptake. The results demonstrate that CD@EXO_MSC_ effectively triggers tumor-suppressive and oxidative stress-related mechanisms. These exosomes therefore achieve significant improvements over free Chl-CDs. Building on these findings, future research could investigate the applicability of this strategy across various cancer types, including more complex models such as glioblastoma. Additionally, exploring the potential of loading other therapeutic agents or nanomaterials into exosomes might open new avenues for highly targeted and personalized cancer therapies. To translate this promising technology into routine clinical application, addressing the scalability and standardization of exosome production will be critical.

## 5 Methods

### 5.1 Preparation of CD@EXO_MSC_

#### 5.1.1 Internalization of Chl-CDs by Human Placental Mesenchymal Stem Cells

Here, we utilized chlorophyllin-based Carbon Dots (Chl-CDs), which were well characterized in terms of their photodynamic therapy properties in our previous study. Briefly, Chl-CDs were synthesized from chlorophyllin sodium copper salt using a microwave-assisted bottom-up approach.^21^

To evaluate the internalization of Chl-CDs, human placental mesenchymal stem cells (hpMSCs) were seeded at a density of 30,000 cells/well and incubated for 24 h to allow surface attachment. After being treated with 250 μg/mL Chl-CDs at different time points, cells were washed three times with PBS to remove noninternalized nanomaterials. Cells then were collected by trypsinization, counted, and centrifuged at 300*g* for 4 min. Cell pellets were then digested overnight with aqua regia, then filtered through a 0.22 μm filter. To monitorize Chl-CDs internalization, the Cu content of the Chl-CDs was leveraged, by analyzing the content of copper in cells by ICP-MS. Additionally, to confirm the internalization of Chl-CDs, flow cytometry was employed using the optical properties of the Chl-CDs. To this end, hpMSCs were seeded at a density of 150,000 cells per well in 12-well cell culture plates and incubated with the desired concentration of Chl-CDs for 24 h to allow surface attachment. The supernatant was discarded, and the cells were treated with 50 μg Chl-CDs/mL. The cell pellet was recovered as previously described, resuspended in PBS, and analyzed. The intrinsic fluorescence intensities of Chl-CDs within the cells were measured with flow cytometry recording 50,000 events.

#### 5.1.2 Isolation of CD@EXO_MSC_

To obtain CD@EXO_MSC_, hpMSC at 60–70% confluency was treated with complete medium containing 200 μg/mL Chl-CDs for 4 h. After incubation, the cells were washed three times with DPBS to remove noninternalized Chl-CDs. Subsequently, both control and treated groups were reincubated with fresh complete medium containing exosome-free FBS for 48 h. Afterward, the medium was collected, and a serial centrifugation protocol was used for exosome isolation. The supernatant was centrifuged sequentially at 600*g* for 15 min, 2000*g* for 30 min, and 12,000*g* for 30 min to separate the large microvesicles from exosomes. The final supernatant was transferred to ultracentrifuge tubes (cat. No: 355622, Beckman Coulter) and centrifuged at 100,000*g* for 2 h at 4 °C (Beckman Coulter, Optima XL-100). The supernatant was carefully aspirated, and the exosome pellets were collected with ice-cold DPBS, then centrifuged again at 100,000*g* for 2 h at 4 °C. The purified exosomes were stored in DPBS at −80 °C until further use.

### 5.2 Characterization of CD@EXO_MSC_

#### 5.2.1 Protein Quantification

The total protein of the exosomes was determined with a Pierce BCA Protein Assay Kit (cat. No.: 23225, Thermo Scientific). According to the manufacturer’s instructions, a working solution was prepared by mixing of reagents A and B in a 50:1 ratio. The working solution was then distributed into a 96-well culture plate at 100 μL per well. Exosome samples (5 μL) were added to the wells containing the working solution and gently mixed. The plate was covered with aluminum foil and incubated at 37 °C for 30 min. After incubation, absorbance was measured at 562 nm with a plate reader (Synergy HT, Bio-Tek) with Gen 5 software.

#### 5.2.2 Surface Marker Analysis

A MACSPlex EV Kit (cat. No.: 130.122.209) was used to assess the expression of surface markers on exosomes by flow cytometry. According to the supplier’s protocol, 15 μL latex beads and 120 μL exosomes were added to 500 μL kit solution and incubated overnight. The mixture was centrifuged at 3000*g* for 5 min, and the supernatant was carefully aspirated. The pellet containing bead-bound exosomes was resuspended in 500 μL kit solution, and 15 μL CD9, CD63, and CD81 antibody cocktail was added for a 1-h incubation. Afterward, the solution was removed by centrifugation at 3000*g* for 5 min. The exosomes were resuspended in 500 μL kit solution and analyzed using Flow Cytometrywith flow cytometry, recording 50,000 events.

#### 5.2.3 Nanoparticle Tracking Analysis

Nanoparticle tracking analysis (NTA; NS300, Malvern Panalytical, UK) was used to analyze the size distribution of exosomes. The isolated exosomes were diluted in PBS at a ratio of 1:100 and loaded into the device with capillary tubes. The flow rate was sequentially adjusted to 5, 20, 40, and 60 μL/min to advance the exosomes to the camera recording area. After recording began, the flow rate was fixed at 5 μL/min, and approximately 1000 particles were measured within 60 s.

#### 5.2.4 Transmission Electron Microscopy Characterization

For morphological analysis of the isolated exosomes, transmission electron microscopy (TEM) was performed using a FEI TECNAI T20 microscope operated at 200 keV. Exosomes in DPBS were dropcasted on a TEM nickel grid and allowed to adsorb at room temperature for 20 min. The grids were then washed with a drop of deionized water for 1 min, and excess water was absorbed with a piece of paper. To provide contrast, 5 μL 1% uranyl acetate was applied to the exosome-coated surface of the grid and allowed to air-dry at room temperature, and microscopy imaging was performed.

#### 5.2.5 Analysis of Cu Content in Exosomes by HAADF-STEM and EDX Mapping Analysis

Aberration corrected scanning transmission electron microscopy (Cs-corrected STEM) images were acquired using a high angle annular dark field detector in a FEI XFEG TITAN electron microscope (Hillsboro, Oregon) operated at 300 kV and equipped with a CETCOR Cs-probe corrector from CEOS GmbH (Heidelberg, Germany), allowing the formation of an electron probe of 0.08 nm. The geometric aberrations of the probe-forming system were controlled to allow a beam convergence of 24.7 mrad half an gle. Elemental analysis was carried out with an EDX detector for EDS experiments in scanning mode. EDX mappings were acquired with an Oxford Instruments (NanoAnalysis & Asylum Research, High Wycombe, UK) detector and analyzed with the built-in AZtec software. Energy dispersive X-ray (EDX) analysis was performed to detect copper originating from Chl-CDs in the exosome content, and high-resolution transmission electron microscopy (HR-TEM) was used to identify the crystalline structure of Chl-CDs within the exosomes. In this case, a Titan FEI TITAN, operated at 300 kV and equipped with a Gatan Image Filter (GIF Tridiem 863). To minimize signal contamination in elemental analyses, exosomes were collected in deionized water instead of PBS during the final stage of isolation. After exosomes were transferred onto nickel grids, they were allowed to dry at room temperature for 20 min. No fixatives or contrast staining agents were used, to prevent potential crossed contamination.

#### 5.2.6 Exosome Loading Efficiency

To quantify the amounts of Chl-CDs carried by exosomes, spectrophotometric measurements were performed. Free Chl-CDs serial dilutions (0–1000 μg/mL) were distributed into 100 μL aliquots in 96-well culture plates, and the absorbance was measured at 400 nm to generate a calibration curve. The absorbance value obtained from Chl-CDs containing exosomes (CD@EXO_MSC_) was used to calculate the amount of Chl-CDs according to the Chl-CDs standard calibration curve. The amount of Chl-CDs per microgram of exosome was then calculated.

### 5.3 Exosome-Mediated PDT Treatment In Vitro

#### 5.3.1 Cytotoxicity Assay

To determine the optimal working dose for photodynamic therapy, a noncytotoxic dose of exosomes was established. A549 (human lung adenocarcinoma), U87-MG (human glioblastoma multiforme), and U251-MG (human glioblastoma) cancer cells were seeded into 96-well cell culture plates at a density of 5,000 cells per well and incubated under standard conditions for 24 h to allow the cells to attach. Subsequently, exosomes were added to the cancer cells (2.5, 5, and 10 μg/mL) in 100 μL and incubated for 4 h, to select the optimal concentration for PDT treatment. For in vitro assessment of exosome-mediated PDT, cancer cells were seeded into 96-well culture plates and, after 24 h, were treated with 5 μg/mL exosomes for 4 h. After incubation, the cells were washed three times and irradiated with 520 nm light for 1 h. After the treatments, cell viability was assessed with LDH assays (CyQUANT LDH Cytotoxicity Assay, cat. No.: C20300, Thermo Fisher Scientific).

#### 5.3.2 Uptake of Exosomes by Cancer Cells

Exosome uptake by cancer cells was evaluated according to the intrinsic fluorescence of Chl-CDs. U87-MG, A549, and U251-MG cell lines were seeded at a density of 150,000 cells per well in 12-well cell culture plates. After 24 h of incubation to allow surface attachment, the supernatant was aspirated and completed medium containing either 5 μg/mL empty mesenchymal stem cell exosomes (EXO_MSC_) or CD@EXO_MSC_ was added to the respective wells for 4 h. After incubation, the supernatant was aspirated, and the cells were washed three times with DPBS to remove noninternalized exosomes. The cells were trypsinized and then centrifuged at 300*g* for 4 min, and the cell pellet was suspended in 200 μL DPBS. Cells were analyzed on a Novocyte Flow Cytometer with Qdot 655 flourescence filter, recording 50,000 events.

### 5.4 Transcriptomic Assessments

#### 5.4.1 Exosomal miRNA Profiling of EXO_MSC_ and CD@EXO_MSC_

To evaluate the effect of Chl-CD treatment on hpMSC exosome content, we isolated exosomes from hpMSCs incubated with 200 μg/mL Chl-CDs for 4 h, as described above. Exosome isolation was performed 48 h after treatment, and the Exosome Total RNA and Protein Isolation Kit (cat. No.: 4478545, Thermo Fisher Scientific, Inc.) was used for exosomal miRNA extraction.

#### 5.4.2 miRNA Profiling of U87-MG Cancer Cells

To investigate the mechanisms underlying cell death in U87-MG after administration of free Chl-CDs and exosome-mediated PDT, we performed cellular miRNA transcriptome analysis. Cells were seeded into T25 culture flasks at a density of 1 × 10^6^ cells per flask and incubated for 24 h to allow attachment. After aspiration of the medium, the cells were treated with 50 μg/mL free Chl-CDs or 5 μg/mL CD@EXO_MSC_, then incubated for 4 h. After incubation, the cells were washed three times with DPBS, and light-treated groups were irradiated with 520 nm light for 1 h. After PDT treatment, miRNA isolation was performed on all cell groups with a NucleoSpin RNA Mini Purification Kit (Macherey-Nagel GmbH & Co.).

The miRNA sequencing data were processed and analyzed according to a comprehensive bioinformatics workflow. Initially, raw FASTQ files were obtained and subjected to quality control and trimming. Adapter sequences and low-quality reads were removed with Trim Galore (version 0.6.7) (Babraham Bioinformatics), which integrates Cutadapt (version 3.4) and FastQC (version 0.11.9). Post-trimming quality control was performed with multiQC (version 1.11)^63^ to ensure high-quality clean data. The clean reads were then aligned to the reference genome with miRDeep2 (version 2.0.1.2),^64^ which also facilitated the identification and quantification of known and novel miRNAs. The alignment process involved removal of N-reads and polyX sequences to enhance accuracy. Subsequently, miRNA counts were generated with miRDeep2’s quantification module. Differential expression analysis was performed with DESeq2 (version 1.38.1)^65^ to identify differentially expressed genes and normalized counts. Annotation and pathway enrichment analyses were conducted with Diana Tools (version 8.0) and Cytoscape (version 3.10.0),^66^ respectively, to provide insights into the functional implications of the identified miRNAs. Finally, data visualization and further statistical analyses were performed with Python-based libraries, including pandas, numpy, matplotlib, seaborn, and htseq,^67^ facilitated by the bioconda environment.^68^

### 5.5 Exosome-Mediated PDT Treatment In Vivo

#### 5.5.1 Xenograft Cancer Model

Six-week-old female BALB/c nude mice (24–30 g) from Bilkent University’s Experimental Animal Research Unit, Turkey, were used for the exosome-mediated PDT experiments. The study was approved by Bilkent University’s local ethics committee (approval: May 13, 2024; meeting No: 5; file No: 10; decision No: 2024/10).

For the orthotopic cancer model, 3 × 10^6^ U87-MG cancer cells were subcutaneously injected into the animals in a 1:1 ratio of cold Matrigel to PBS. After the tumor volumes (length × width^2^) reached approximately 140 mm^3^, the animals were divided into five groups with no statistically significant differences in average tumor volumes. A dose of 40 μg exosomes in 100 μL PBS was administered via tail vein injection. Twenty-4 h after the injection, the light treatment groups were exposed to 520 nm LED light for 1 h. Tumor sizes were measured every 3–4 days, and the general health status of the animals was monitored.

#### 5.5.2 Exosome Biodistribution and Biosafety

For assessment of the exosomes’ biodistribution, the intrinsic fluorescence of Chl-CDs was analyzed. A total of 40 μg exosomes in 100 μL PBS was administered via tail vein injection. Biodistribution was assessed 24 h postinjection with an IVIS imaging system (PerkinElmer). Balb/c mice were intravenously injected via the tail vein with 40 μg/mL CD@EXO_MSC_ or 250 μg/mL Chl-CD. Blood samples were collected on days 1, 7, and 14 using yellow-top biochemistry tubes, and serum levels of ALT, ALP, AST, urea, and creatinine were quantified. To evaluate the structural stability of CD@EXO_MSC_, NTA measurements were performed following incubation in exosome-free FBS.

#### 5.5.3 Histology

The animals were sacrificed by cervical dislocation, and their organs were fixed in 10% neutral buffered formalin solution for 72 h for histological examination. Serial sections of 5 μm thickness were obtained from paraffin-embedded blocks with a microtome, and standard hematoxylin-eosin staining was performed. Immunohistochemical (IHC) staining was performed on serial tissue sections using the standard streptavidin–biotin complex technique. Following deparaffinization and rehydration, endogenous peroxidase activity was blocked by incubating the sections in a 3% hydrogen peroxide solution for 15 min. Heat-induced epitope retrieval was then performed using a sodium citrate buffer (10 mM, pH 6), which facilitated the breaking of methylene bridges between proteins. Afterward, the sections were incubated with 10% normal rabbit serum for 30 min to block nonspecific binding sites. Subsequently, the sections were exposed to primary antibodies for PCNA (1:1000, MAB424, EMD Millipore, USA) and Caspase 3 (1:50, 9661, Cell Signalingg, USA) overnight. After washing with TBS, the sections were incubated with biotinylated antirabbit antibody (IgG BA1000, Vector Laboratories Inc., CA, USA) for 30 min. Another wash step followed, and the sections were then treated with peroxidase- conjugated streptavidin (Standard Vectastain Elite ABC Kit, PK-6100, Vector Laboratories Inc., CA, USA) for 30 min. The reaction was visualized by incubating the sections with DAB (3,3′–diaminobenzidine substrate, SK-4100, Vector Laboratories Inc., CA, USA). Finally, the slides were counterstained with Gill’s (III) hematoxylin and mounted with coverslips. The stained sections were examined under a light microscope (DM2500, Leica, Wetzlar, Germany) and images were captured using a digital camera (DFC450, Leica, Wetzlar, Germany).

#### 5.5.4 Statistics

For statistical analyses, GraphPad Prism software version 8.4.3 (471) was used. Two-way ANOVA followed by Tukey’s multiple comparison test was conducted, with *p* < 0.05 considered statistically significant (0.1234 (ns); 0.0332 (*); 0.0021 (**); 0.0002 (***); < 0.0001 (****)).To compare the five groups, one-way ANOVA was used separately for PCNA and Caspase 3 staining intensities. Tukey’s post hoc test was applied to determine pairwise differences between groups. A *p*-value of less than 0.05 was considered statistically significant. Statistical significance is indicated as follows: * < 0.05, ** ≤ 0.01, *** ≤ 0.001, **** ≤ 0.0001. All experiments were performed on at least three technical and three biological replicates

## Supplementary Material



## Data Availability

Data will be made available on reasonable request.

## Notes

Detailed characterization of carbon dots; additional experiments, biodistribution, stability, and biosafety analyses of exosomes; supplementary transcriptomic data; and the list of miRNAs and genes associated with the underlying mechanisms (PDF)

## References

[1] Soltanmohammadi F., Gharehbaba A. M., Zangi A. R., Adibkia K., Javadzadeh Y. (2024). Current knowledge
of hybrid nanoplatforms composed of exosomes and organic/inorganic
nanoparticles for disease treatment and cell/tissue imaging. Biomedicine & Pharmacotherapy.

[2] Sharma V., Mukhopadhyay C. D. (2024). Exosome
as drug delivery system: Current advancements. Extracellular Vesicle.

[3] Lara P., Palma-Florez S., Salas-Huenuleo E., Polakovicova I., Guerrero S., Lobos-Gonzalez L., Campos A., Muñoz L., Jorquera-Cordero C., Varas-Godoy M. (2020). Gold nanoparticle based
double-labeling of melanoma extracellular vesicles to determine the
specificity of uptake by cells and preferential accumulation in small
metastatic lung tumors. J. Nanobiotechnol..

[4] Kim M. S., Haney M. J., Zhao Y., Mahajan V., Deygen I., Klyachko N. L., Inskoe E., Piroyan A., Sokolsky M., Okolie O. (2016). Development
of exosome-encapsulated paclitaxel to overcome
MDR in cancer cells. Nanomedicine.

[5] Catalano A., Iacopetta D., Ceramella J., Scumaci D., Giuzio F., Saturnino C., Aquaro S., Rosano C., Sinicropi M. S. (2022). Multidrug
resistance (MDR): A widespread phenomenon in pharmacological therapies. Molecules.

[6] Zhao C., Qiu L., Wu D., Zhang M., Xia W., Lv H., Cheng L. (2023). Targeted reversal of multidrug resistance
in ovarian cancer cells
using exosome-encapsulated tetramethylpyrazine. Mol. Med. Rep..

[7] Sancho-Albero M., Sebastian V., Perez-Lopez A. M., Martin-Duque P., Unciti-Broceta A., Santamaria J. (2024). Extracellular Vesicles-Mediated Bio-Orthogonal
Catalysis in Growing Tumors. Cells.

[8] Sancho-Albero M., Martín-Pardillos A., Lujan L., Sebastian V., Santamaria J., Martín-Duque P. (2022). Exosomes loaded with ultrasmall Pt
nanoparticles: a novel low-toxicity alternative to cisplatin. J. Nanobiotechnol..

[9] Sancho-Albero M., Encinas-Giménez M., Sebastián V., Pérez E., Luján L., Santamaría J., Martin-Duque P. (2022). Transfer of photothermal nanoparticles
using stem cell
derived small extracellular vesicles for in vivo treatment of primary
and multinodular tumours. J. Extracell. Vesicles.

[10] Wang J., Fu Y., Gu Z., Pan H., Zhou P., Gan Q., Yuan Y., Liu C. (2024). Multifunctional
carbon dots for biomedical
applications: diagnosis, therapy, and theranostic. Small.

[11] Madrid A., Martín-Pardillos A., Bonet-Aleta J., Sancho-Albero M., Martinez G., Calzada-Funes J., Martin-Duque P., Santamaria J., Hueso J. L. (2023). Nitrogen-doped carbon
nanodots deposited on titania nanoparticles: Unconventional near-infrared
active photocatalysts for cancer therapy. Catal.
Today.

[12] Yu Y., Zeng Q., Tao S., Xia C., Liu C., Liu P., Yang B. (2023). Carbon dots based photoinduced
reactions: advances
and perspective. Adv. Sci..

[13] Madrid A., Martinez G., Hornos F., Bonet-Aleta J., Calvo E., Lozano A., Hueso J. L. (2023). Laser-induced tuning
of carbon nanosensitizers to maximize nitrogen doping and reactive
oxygen species production in the visible range. Catal. Today.

[14] Edis Z., Wang J., Waqas M. K., Ijaz M., Ijaz M. (2021). Nanocarriers-mediated
drug delivery systems for anticancer agents: an overview and perspectives. Int. J. Nanomed..

[15] Tiwari P., Shukla R. P., Yadav K., Singh N., Marwaha D., Gautam S., Bakshi A. K., Rai N., Kumar A., Sharma D. (2024). Dacarbazine-primed carbon quantum dots coated with
breast cancer cell-derived exosomes for improved breast cancer therapy. J. Controlled Release.

[16] Li J., Kong J., Ma S., Li J., Mao M., Chen K., Chen Z., Zhang J., Chang Y., Yuan H. (2021). Exosome-coated ^10^B carbon dots for precise
boron neutron capture therapy in a mouse model of glioma in situ. Adv. Funct. Mater..

[17] Rupaimoole R., Slack F. J. (2017). MicroRNA therapeutics: towards a new era for the management
of cancer and other diseases. Nat. Rev. Drug
Discovery.

[18] Yilmazer A., de Lázaro I., Taheri H. (2015). Reprogramming cancer cells: a novel
approach for cancer therapy or a tool for disease-modeling?. Cancer Letters.

[19] Hannafon B. N., Ding W.-Q. (2013). Intercellular communication
by exosome-derived microRNAs
in cancer. International journal of molecular
sciences.

[20] Li C., Zhou T., Chen J., Li R., Chen H., Luo S., Chen D., Cai C., Li W. (2022). The role of Exosomal
miRNAs in cancer. J. Transl. Med..

[21] Kirbas
Cilingir E., Besbinar O., Giro L., Bartoli M., Hueso J. L., Mintz K. J., Aydogan Y., Garber J. M., Turktas M., Ekim O. (2024). Small Warriors of Nature:
Novel Red Emissive Chlorophyllin Carbon Dots Harnessing Fenton-Fueled
Ferroptosis for In Vitro and In Vivo Cancer Treatment. Small.

[22] de
Araujo Farias V., O’Valle F., Serrano-Saenz S., Anderson P., Andrés E., López-Peñalver J., Tovar I., Nieto A., Santos A., Martín F. (2018). Exosomes derived from mesenchymal stem cells enhance radiotherapy-induced
cell death in tumor and metastatic tumor foci. Mol. Cancer.

[23] Fath M. K., Anjomrooz M., Taha S. R., Zadeh M. S., Sahraei M., Atbaei R., Naghibi A. F., Payandeh Z., Rahmani Z., Barati G. (2022). The therapeutic
effect of exosomes from mesenchymal
stem cells on colorectal cancer: Toward cell-free therapy. Pathol. Res. Pract..

[24] More D. A., Singh N., Mishra R., Muralidharan H. P., Gopinath K. S., Gopal C., Kumar A. (2024). Intronic miR-6741–3p
targets the oncogene SRSF3: Implications for oral squamous cell carcinoma
pathogenesis. PLoS One.

[25] Yan Z., Hong S., Song Y., Bi M. (2021). microR-4449 promotes
colorectal cancer cell proliferation via regulation of SOCS3 and activation
of STAT3 signaling. Cancer Manage. Res..

[26] Chen L., Li Z., Hu S., Deng Q., Hao P., Guo S. (2022). Extracellular
vesicles carry miR-27a-3p to promote drug resistance of glioblastoma
to Temozolomide by targeting BTG2. Cancer Chemotherapy
and Pharmacology.

[27] Zhang J., Cao Z., Yang G., You L., Zhang T., Zhao Y. (2019). MicroRNA-27a
(miR-27a) in solid tumors: a review based on mechanisms and clinical
observations. Front. Oncol..

[28] Datta, D. ; Bhattacharyya, S. N. Oxidative Stress-Mediated miRNA Regulation in Cancer. In Handbook of Oxidative Stress in Cancer: Therapeutic Aspects; Springer, 2022; pp 1339–1355.

[29] Li Z., Pei Q., Zheng Y., Xie Z., Zheng M. (2023). Carbon dots for long-term
near-infrared afterglow imaging and photodynamic therapy. Chemical Engineering Journal.

[30] Kuznietsova H., Géloën A., Dziubenko N., Zaderko A., Alekseev S., Lysenko V., Skryshevsky V. (2023). In vitro and in vivo toxicity of
carbon dots with different chemical compositions. Discover Nano.

[31] Sousa D. A., Ferreira L. F., Fedorov A. A., Rego A. M. d., Ferraria A. M., Cruz A. B., Berberan-Santos M. N., Prata J. V. (2022). Luminescent carbon
dots from wet olive pomace: structural insights, photophysical properties
and cytotoxicity. Molecules.

[32] Xu D., Guo D., Zhang J., Tan X., Deng Z., Hou X., Wang S. (2024). Innovative tumor interstitial
fluid-triggered carbon dot-docetaxel
nanoassemblies for targeted drug delivery and imaging of HER2-positive
breast cancer. Int. J. Pharm..

[33] Qiao L., Xuan W., Ou Y., Li L., Wu S., Guo Y., Liu M., Yu D., Chen Q., Yuan J. (2024). Tumor microenvironment
activation amplify oxidative stress promoting
tumor energy remodeling for mild photothermal therapy and cuproptosis. Redox Biol..

[34] Kazeminava F., Javanbakht S., Latifi Z., Rasoulzadehzali M., Abbaszadeh M., Alimohammadzadeh B., Mahdipour M., Fattahi A., Hamishehkar H., Adibag Z. (2024). Ultrasound-assisted
encapsulating folic acid-based carbon quantum dots within breast cancer
cell-derived exosomes as a co-receptors-mediated anticancer nanocarrier
for enhanced breast cancer therapy. Sci. Rep..

[35] Cheng Z., Que H., Chen L., Sun Q., Wei X. (2022). Nanomaterial-based
drug delivery system targeting lymph nodes. Pharmaceutics.

[36] Fu S., Wang Y., Xia X., Zheng J. C. (2020). Exosome engineering:
Current progress in cargo loading and targeted delivery. NanoImpact.

[37] Sancho-Albero M., del Mar Encabo-Berzosa M., Beltran-Visiedo M., Fernandez-Messina L., Sebastian V., Sanchez-Madrid F., Arruebo M., Santamaria J., Martin-Duque P. (2019). Efficient
encapsulation of theranostic nanoparticles in cell-derived exosomes:
leveraging the exosomal biogenesis pathway to obtain hollow gold nanoparticle-hybrids. Nanoscale.

[38] Hoshino A., Costa-Silva B., Shen T.-L., Rodrigues G., Hashimoto A., Tesic Mark M., Molina H., Kohsaka S., Di Giannatale A., Ceder S. (2015). Tumour exosome integrins
determine organotropic metastasis. Nature.

[39] Sancho-Albero M., Navascués N., Mendoza G., Sebastián V., Arruebo M., Martín-Duque P., Santamaría J. (2019). Exosome origin
determines cell targeting and the transfer of therapeutic nanoparticles
towards target cells. J. Nanobiotechnol..

[40] Padinharayil H., Varghese J., Wilson C., George A. (2024). Mesenchymal stem cell-derived
exosomes: characteristics and applications in disease pathology and
management. Life Sciences.

[41] Kim H., Kim E. H., Kwak G., Chi S.-G., Kim S. H., Yang Y. (2021). Exosomes: cell-derived
nanoplatforms for the delivery of cancer therapeutics. International journal of molecular sciences.

[42] Bu W., Xu X., Wang Z., Jin N., Liu L., Liu J., Zhu S., Zhang K., Jelinek R., Zhou D. (2020). Ascorbic
acid-PEI carbon dots with osteogenic effects as miR-2861 carriers
to effectively enhance bone regeneration. ACS
Appl. Mater. Interfaces.

[43] Zhao R., Chen X., Song H., Bie Q., Zhang B. (2020). Dual role
of MSC-derived exosomes in tumor development. Stem Cells Int..

[44] Gupta D., Zickler A. M., El Andaloussi S. (2021). Dosing extracellular vesicles. Advanced drug delivery reviews.

[45] Li J., Kong C., Song W., Fu T. (2023). Identification of cuproptosis-related
subtypes, establishment of a prognostic signature and characterization
of the tumor microenvironment in gastric cancer. Int. J. Gen. Med..

[46] Wu J.-h., Cheng T.-c., Zhu B., Gao H.-y., Zheng L., Chen W.-x. (2023). Identification of
cuproptosis-related gene SLC31A1
and upstream LncRNA-miRNA regulatory axis in breast cancer. Sci. Rep..

[47] Wang H., Yang Z., He X., Guo F., Sun H., Xu S., Xu C., Wang Z., Wen H., Teng Z. (2023). Cuproptosis related gene PDHB is identified
as a biomarker inversely
associated with the progression of clear cell renal cell carcinoma. BMC Cancer.

[48] Qi Y., Yao Q., Li X., Li X., Zhang W., Qu P. (2023). Cuproptosis-related
gene SLC31A1: prognosis values and potential biological functions
in cancer. Sci. Rep..

[49] Li Y., Du Y., Zhou Y., Chen Q., Luo Z., Ren Y., Chen X., Chen G. (2023). Iron and copper: critical executioners
of ferroptosis, cuproptosis and other forms of cell death. Cell Commun. Signal..

[50] Bonet-Aleta J., Sancho-Albero M., Calzada-Funes J., Irusta S., Martin-Duque P., Hueso J. L., Santamaria J. (2022). Glutathione-Triggered catalytic response
of Copper-Iron mixed oxide Nanoparticles. Leveraging tumor microenvironment
conditions for chemodynamic therapy. J. Colloid
Interface Sci..

[51] Xiong Y., Xiao C., Li Z., Yang X. (2021). Engineering
nanomedicine
for glutathione depletion-augmented cancer therapy. Chem. Soc. Rev..

[52] Bonet-Aleta J., Alegre-Requena J. V., Martin-Martin J., Encinas-Gimenez M., Martín-Pardillos A., Martin-Duque P., Hueso J. L., Santamaria J. (2024). Nanoparticle-Catalyzed
Transamination
under Tumor Microenvironment Conditions: A Novel Tool to Disrupt the
Pool of Amino Acids and GSSG in Cancer Cells. Nano Lett..

[53] Zang X., Jiang J., Gu J., Chen Y., Wang M., Zhang Y., Fu M., Shi H., Cai H., Qian H. (2022). Circular RNA EIF4G3 suppresses gastric cancer
progression
through inhibition of β-catenin by promoting δ-catenin
ubiquitin degradation and upregulating SIK1. Mol. Cancer.

[54] Mossakowska B. J., Shahmoradi Ghahe S., Cysewski D., Fabisiewicz A., Tudek B., Siedlecki J. A. (2022). Mechanisms
of resistance to photodynamic
therapy (PDT) in vulvar cancer. International
journal of molecular sciences.

[55] Saha S. (2024). Role of microRNA
in oxidative stress. Stresses.

[56] Bu H., Wedel S., Cavinato M., Jansen-Dürr P. (2017). MicroRNA regulation
of oxidative stress-induced cellular senescence. Oxid. Med. Cell. Longevity.

[57] Hwang T. I.-S., Chen P.-C., Tsai T.-F., Lin J.-F., Chou K.-Y., Ho C.-Y., Chen H.-E., Chang A.-C. (2022). Hsa-miR-30a-3p overcomes
the acquired protective autophagy of bladder cancer in chemotherapy
and suppresses tumor growth and muscle invasion. Cell Death Dis..

[58] Feng L., Jing W., Jin S., Wang B. (2023). Circ_0088194 regulates
proliferation, migration, apoptosis, and inflammation by miR-30a-3p/ADAM10
axis in rheumatoid arthritis fibroblastic synovial cells. Inflammation.

[59] Matson K., Macleod A., Mehta N., Sempek E., Tang X. (2023). Impacts of
microRNA-483 on human diseases. Non-coding RNA.

[60] Moscetti I., Cannistraro S., Bizzarri A. R. (2019). Probing direct interaction of oncomiR-21–3p
with the tumor suppressor p53 by fluorescence, FRET and atomic force
spectroscopy. Arch. Biochem. Biophys..

[61] Ju Q., Zhao L., Gao J., Zhou L., Xu Y., Sun Y., Zhao X. (2019). Mutant p53
increases exosome-mediated transfer of miR-21–3p
and miR-769–3p to promote pulmonary metastasis. Chinese Journal of Cancer Research.

[62] Liu F., Di Wang X. (2019). miR-150–5p represses
TP53 tumor suppressor gene
to promote proliferation of colon adenocarcinoma. Sci. Rep..

[63] Ewels P., Magnusson M., Lundin S., Käller M. (2016). MultiQC: Summarize
analysis results for multiple tools and samples in a single report. Bioinformatics.

[64] Friedländer M. R., Mackowiak S. D., Li N., Chen W., Rajewsky N. (2012). miRDeep2 accurately
identifies known and hundreds of novel microRNA genes in seven animal
clades. Nucleic acids research.

[65] Love M. I., Huber W., Anders S. (2014). Moderated
estimation of fold change
and dispersion for RNA-seq data with DESeq2. Genome Biol..

[66] Franz M., Lopes C. T., Fong D., Kucera M., Cheung M., Siper M. C., Huck G., Dong Y., Sumer O., Bader G. D. (2023). Cytoscape. js 2023
update: a graph theory library for
visualization and analysis. Bioinformatics.

[67] Anders S., Pyl P. T., Huber W. (2015). HTSeq—a
Python framework to
work with high-throughput sequencing data. bioinformatics.

[68] Grüning B., Dale R., Sjödin A., Chapman B. A., Rowe J., Tomkins-Tinch C. H., Valieris R., Köster J., Team B. (2018). Bioconda: sustainable and comprehensive software distribution for
the life sciences. Nat. Methods.

