# Supplementary material for: Cell-Driven Encapsulation of Chlorophyllin-Based Carbon Dots within Exosomes for Enhanced Photodynamic Therapy: miRNA Profiling Reveals Mechanistic Insights
Source: ACS Appl Mater Interfaces. 2025 Dec 1;17(49):66434–49. doi: 10.1021/acsami.5c18555 (PMC12874207; doi:10.1021/acsami.5c18555)
Supplement: Supplementary file 1 [file am5c18555_si_001.pdf]

Supporting Information

Cell-Driven Encapsulation of Chlorophyllin-based Carbon Dots within Exosomes for Enhanced Photodynamic Therapy: miRNA Profiling Reveals Mechanistic Insights

Omur Besbinar [1,9], Recep Uyar [1,9,11], Emel Kirbas Cilingir [2], Ana Martín-Pardillos [3,4,5,6], Jose L. Hueso [3,4,5,6,7], Ahmet Ceylan [10], Ozge Ozgenc [10], Okan Ekim [11], Mehmet Altay Unal [1], Fikret Ari [12], Roger M. Leblanc [2], Jesus Santamaria [3,4,5,6], Acelya Yilmazer [1,8] *

[1] Stem Cell Institute, Ankara University, 06520 Ankara, Turkey

[2] Department of Chemistry, University of Miami, 1301 Memorial Drive, Coral Gables, Florida 33146, United States

[3] Instituto de Nanociencia y Materiales de Aragon (INMA); CSIC-Universidad de Zaragoza, Campus Rio Ebro, Edificio I+D, C/ Poeta Mariano Esquillor, s/n, 50018, Zaragoza, Spain

[4] Department of Chemical Engineering and Environmental Technology (IQTMA), University of Zaragoza, 50018 Zaragoza, Spain.

[5] Networking Research Center in Biomaterials, Bioengineering and Nanomedicine (CIBER-BBN), Instituto de Salud Carlos III; 28029 Madrid, Spain.

[6] Instituto de Investigación Sanitaria (IIS) de Aragón, Avenida San Juan Bosco, 13, 50009 Zaragoza, Spain.

[7] Escuela Politécnica Superior, Universidad de Zaragoza, Crta. de Cuarte s/n, 22071, Huesca, Spain

[8] Department of Biomedical Engineering, Faculty of Engineering, Ankara University, 06830 Ankara, Turkey

[9] The Graduate School of Health Sciences of Ankara University, 06110, Ankara, Turkey

[10] Department of Histology Embryology, Faculty of Veterinary Medicine, Ankara University, 06110 Ankara, Turkey

[11] Department of Anatomy, Faculty of Veterinary Medicine, Ankara University, 06110 Ankara, Turkey

[12] Department of Electrical Electronic Engineering, Faculty of Engineering, 06830, Ankara, Turkey

*Corresponding author
Dr. Yilmazer:
Address: Department of Biomedical Engineering, Faculty of Engineering, Ankara University, 06830 Ankara, Turkey
Email Address: ayilmazer@ankara.edu.tr

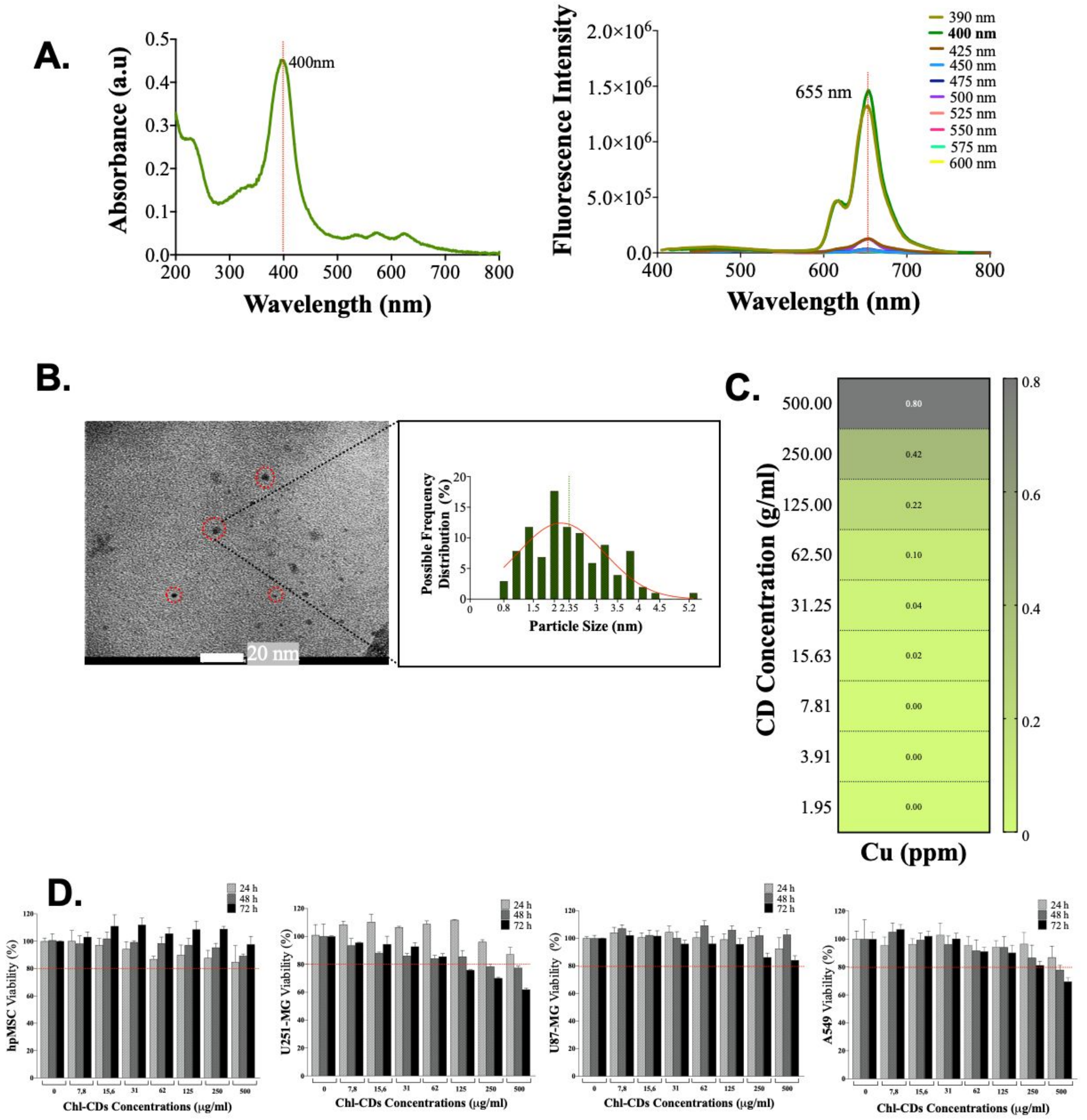

Figure S1: Chlorophyllin-based Carbon Dots Characterization

A. Absorbance and fluorescence measurements of Chl-CDs using UV-VIS and PL spectroscopy, respectively.
B. Morphological visualization and particle size distribution analysis of Chl-CDs using TEM.
C. Measurement of copper content in Chl-CDs using MP-AES.
D. Cytotoxic effect of Chl-CDs in different cell lines.

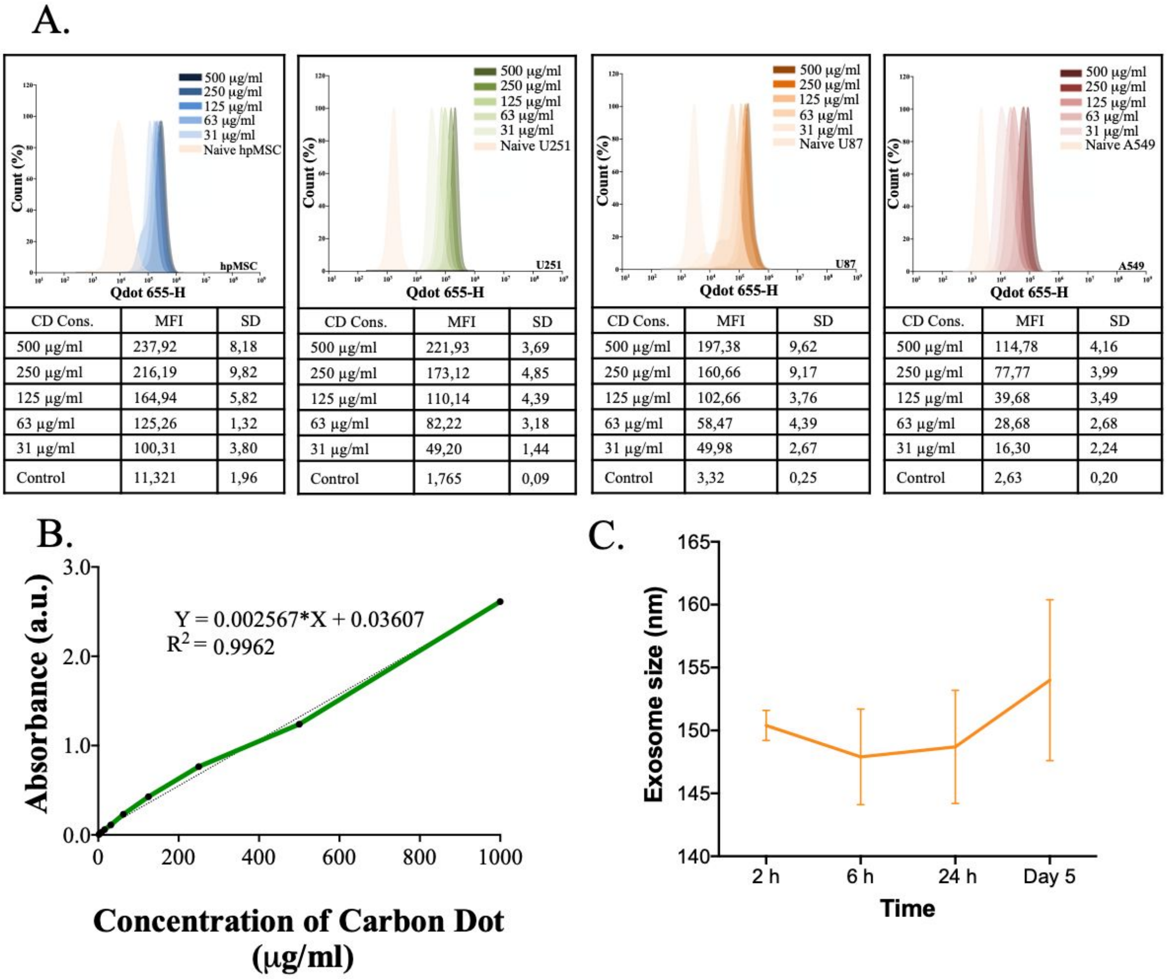

CD Cons.	MFI	SD
500 μg/ml	237,92	8,18
250 μg/ml	216,19	9,82
125 μg/ml	164,94	5,82
63 μg/ml	125,26	1,32
31 μg/ml	100,31	3,80
Control	11,321	1,96

CD Cons.	MFI	SD
500 μg/ml	221,93	3,69
250 μg/ml	173,12	4,85
125 μg/ml	110,14	4,39
63 μg/ml	82,22	3,18
31 μg/ml	49,20	1,44
Control	1,765	0,09

CD Cons.	MFI	SD
500 μg/ml	197,38	9,62
250 μg/ml	160,66	9,17
125 μg/ml	102,66	3,76
63 μg/ml	58,47	4,39
31 μg/ml	49,98	2,67
Control	3,32	0,25

CD Cons.	MFI	SD
500 μg/ml	114,78	4,16
250 μg/ml	77,77	3,99
125 μg/ml	39,68	3,49
63 μg/ml	28,68	2,68
31 μg/ml	16,30	2,24
Control	2,63	0,20

Figure S2: Chlorophyllin-based Carbon Dots Loaded Exosomes

A. Assessment of Chl-CDs internalization at different concentrations and in various cell types by flow cytometry.
B. Absorbance calibration curve of Chl-CDs to calculate the loading efficiency into the exosomes.
C. The stability of exosomes ($CD@EXO_{MSC}$) in fetal bovine serum (FBS).

A.

B.

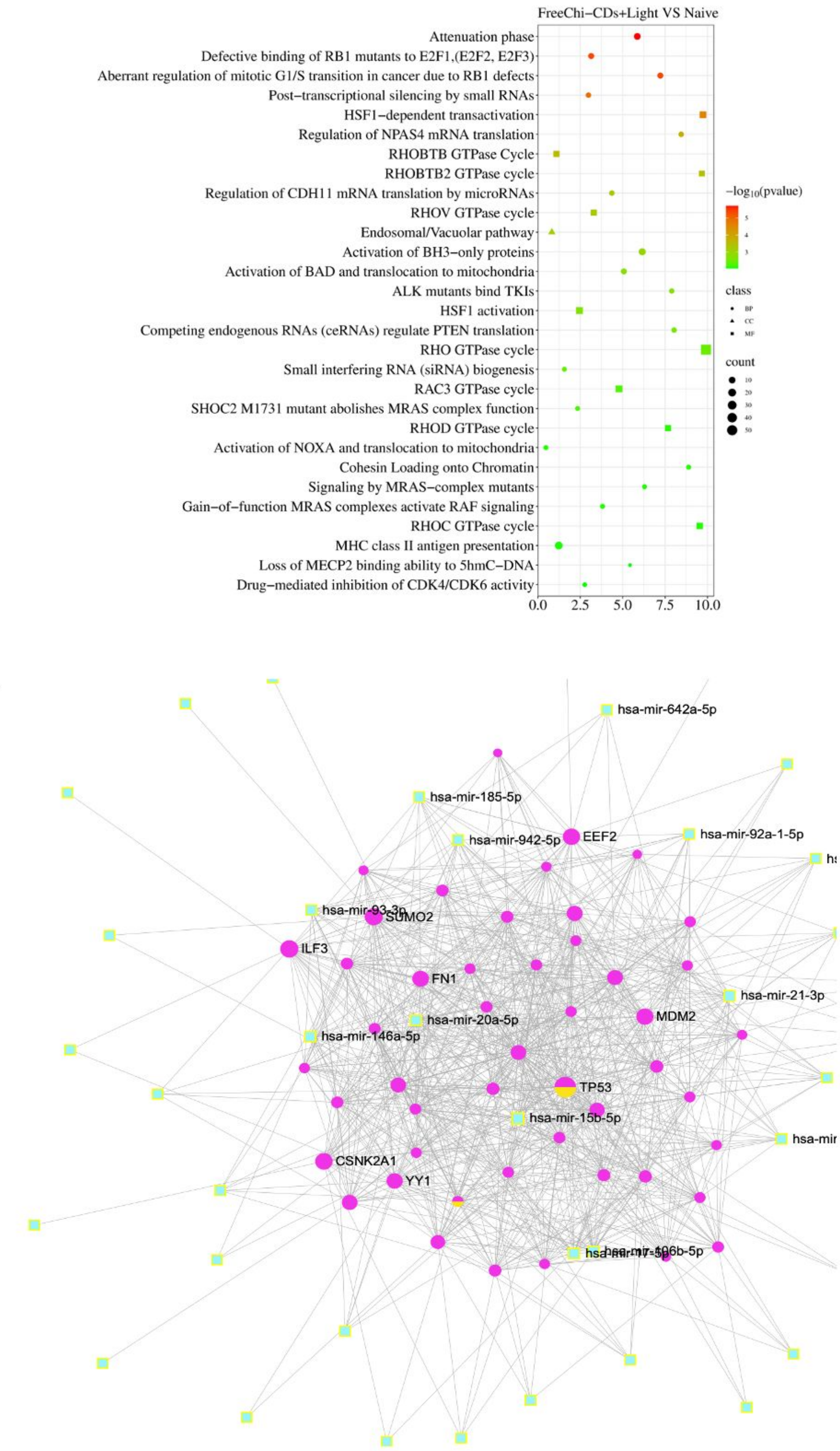

Figure S3. Transcriptomic Analysis of ChI-CD Treated U87 Cells

A. GO Analysis for Free ChI-CDs+LIGHT vs Naive U87 Cells.
B. Network diagram showing interactions between miRNAs and mRNAs, with node size indicating connectivity. Key nodes like TP53 and MDM2 have high connectivity, highlighting their central role in the regulatory network.

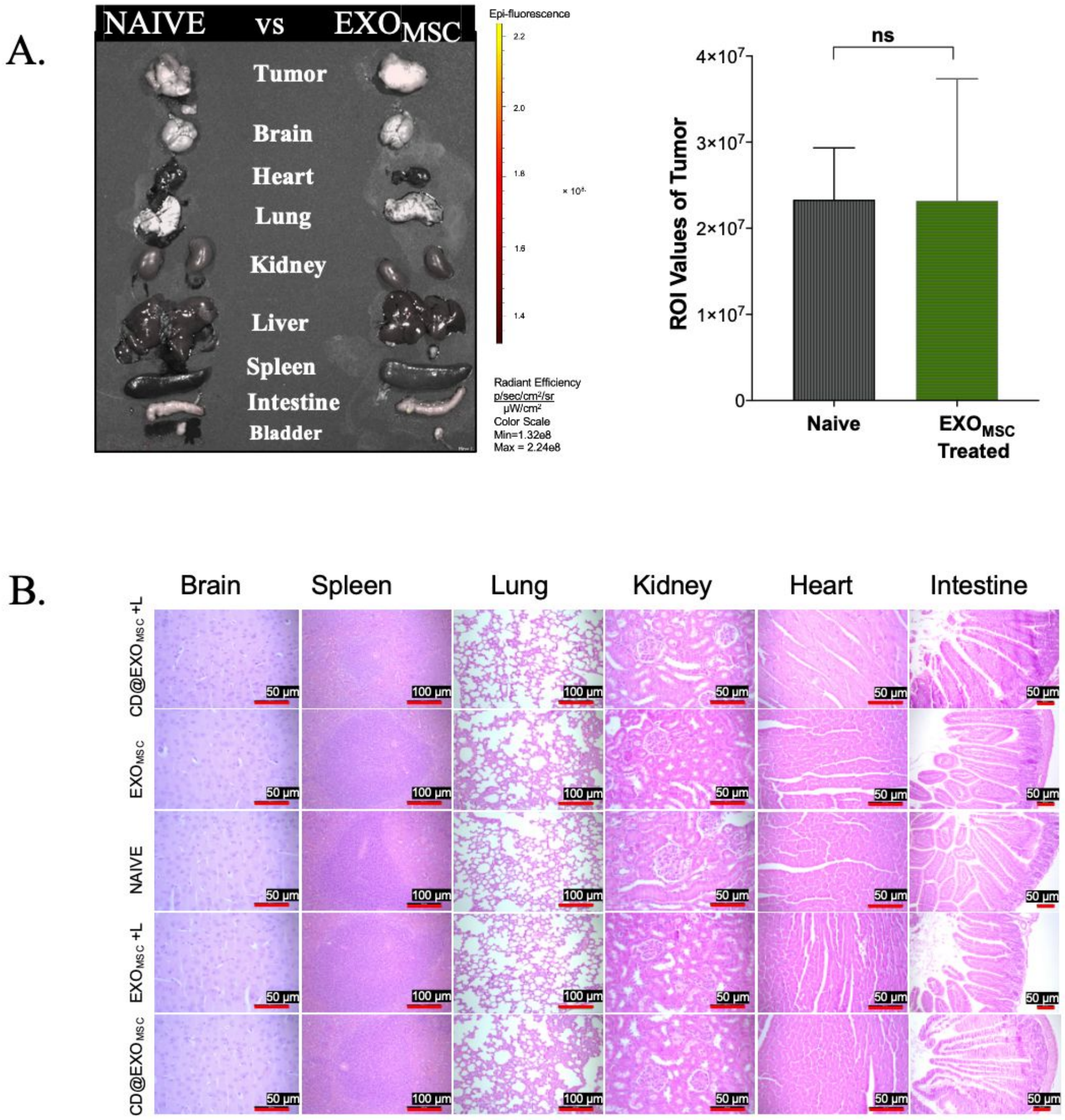

Figure S4. Exosome Biodistribution and Biosafety

A. Biodistribution of empty exosomes in a mouse tumor model using IVIS.
B. Histological images show healthy and normal microstructure in internal organs (H&E stain).

Oncogene-related miRNAs	Endocytosis and Membrane Transport Protein-related miRNAs	miRNAs Involved in Tumor Suppressor Mechanisms	miRNAs Involved in Cancer Resistance Mechanisms	miRNAs Involved in Oxidative Stress Mechanisms
hsa-mir-20a-5p	hsa-mir-15b-5p	hsa-mir-17-5p	hsa-mir-146a-5p	hsa-mir-15b-5p
hsa-mir-15b-5p	hsa-mir-106b-5p	hsa-mir-20a-5p	hsa-mir-15b-5p	hsa-mir-185-5p
hsa-mir-642a-5p	hsa-mir-17-5p	hsa-mir-15b-5p	hsa-mir-17-5p	hsa-mir-17-5p
hsa-mir-1237-3p	hsa-mir-181a-3p	hsa-mir-185-5p	hsa-mir-29b-1-5p	hsa-mir-20a-5p
hsa-mir-1248	hsa-mir-20a-5p	hsa-mir-106b-5p	hsa-mir-93-3p	hsa-mir-106b-5p
hsa-mir-106b-5p	hsa-mir-21-3p	hsa-mir-339-5p	hsa-mir-20a-5p	hsa-mir-181a-3p
hsa-mir-17-5p	hsa-mir-374b-5p	hsa-mir-210-5p	hsa-mir-106b-5p	hsa-mir-21-3p
hsa-mir-21-3p	hsa-mir-632	hsa-mir-619-5p	hsa-mir-339-5p	hsa-mir-324-5p
hsa-mir-374b-5p	hsa-mir-93-3p	hsa-mir-1291	hsa-mir-708-3p	hsa-mir-92a-1-5p
hsa-mir-612	hsa-mir-185-5p	hsa-mir-146a-5p	hsa-mir-185-5p	hsa-mir-146a-5p
hsa-mir-632	hsa-mir-146a-5p	hsa-mir-21-3p	hsa-mir-4521	hsa-mir-612
hsa-mir-185-5p	hsa-mir-642a-5p	hsa-mir-374b-5p	hsa-mir-92a-1-5p	hsa-mir-708-3p
hsa-mir-324-5p	hsa-mir-1246	hsa-mir-5009-5p	hsa-mir-99a-3p	hsa-mir-942-5p
hsa-mir-93-3p	hsa-mir-99a-3p	hsa-mir-99a-3p	hsa-mir-374b-5p	hsa-mir-374b-5p
hsa-mir-942-5p	hsa-mir-342-3p	hsa-mir-194-3p	hsa-mir-663a	hsa-mir-3648
hsa-mir-6511b-3p	hsa-mir-194-3p	hsa-mir-27b-5p	hsa-mir-942-5p	hsa-mir-27b-5p
hsa-mir-4521	hsa-mir-324-5p	hsa-mir-342-3p	hsa-mir-1248	hsa-mir-342-3p
hsa-mir-99a-3p	hsa-mir-4521	hsa-mir-708-3p	hsa-mir-3188	hsa-mir-4521
hsa-mir-4466	hsa-mir-92a-1-5p	hsa-mir-92a-1-5p	hsa-mir-21-3p	hsa-mir-1291
hsa-mir-619-5p	hsa-mir-942-5p	hsa-mir-942-5p	hsa-mir-181a-3p	hsa-mir-93-3p
hsa-mir-146a-5p	hsa-mir-2276-3p	hsa-mir-2276-3p	hsa-mir-342-3p	hsa-mir-339-5p
hsa-mir-24-1-5p	hsa-mir-1244	hsa-mir-181a-3p	hsa-mir-5009-5p	hsa-mir-1244
hsa-mir-339-5p	hsa-mir-708-3p	hsa-mir-29b-1-5p	hsa-mir-194-3p	hsa-mir-99a-3p
hsa-mir-342-3p	hsa-mir-612	hsa-mir-4521	hsa-mir-1291	hsa-mir-642a-5p
hsa-mir-1246	hsa-mir-339-5p	hsa-mir-93-3p	hsa-mir-1246	hsa-mir-29b-1-5p
hsa-mir-5009-5p	hsa-mir-27b-5p	hsa-mir-324-5p	hsa-mir-324-5p	hsa-mir-194-3p
hsa-mir-3648	hsa-mir-4423-5p	hsa-mir-1246	hsa-mir-642a-5p	hsa-mir-1246
hsa-mir-27b-5p	hsa-mir-619-5p	hsa-mir-24-1-5p	hsa-mir-24-1-5p	hsa-mir-24-1-5p
hsa-mir-663b	hsa-mir-4466	hsa-mir-4423-5p	hsa-mir-612	hsa-mir-6511b-3p
hsa-mir-181a-3p		hsa-mir-612	hsa-mir-27b-5p	
hsa-mir-1291		hsa-mir-663a	hsa-mir-4423-5p	
hsa-mir-29b-1-5p				
hsa-mir-92a-1-5p				
hsa-mir-663a				
hsa-mir-194-3p				
hsa-mir-1244				
hsa-mir-210-5p				
hsa-mir-708-3p				
hsa-mir-4423-5p				

Table S1. List of miRNA associated with the mechanisms

Oncogenes	Endocytosis and Membrane Transport Proteins	Genes Involved in Tumor Suppressor Mechanisms	Genes Involved in Cancer Resistance Mechanisms	Genes Involved in Oxidative Stress Mechanisms
ABL2	ATP2A2	BTG2	BAG6	AGPAT1
AGO1	ATP2B1	DICER1	BCL2L11	APP
AGO3	CLTC	MATR3	CDKN1A	BACH1
ARHGAP11A	DYNLL2	NFKBIL1	MDM2	CCND1
BCL2L2	LDLR	PHLPP2	MINK1	CRK
BRWD1	MAPK1	PPARG	NFIB	EEF1D
CCND1	PARD6B	PPP1R18	PRPF8	FEM1B
CCND2	PXK	PTEN	RORA	HSP90AA1
CDK6	RAB11FIP1	RB1	SRCAP	HSPA1A
FNBP4	RAB22A	RBL1	SQSTM1	HSPA1B
G3BP1	SLC1A5	SESN3	TP53	ID3
IGF1R	SLC16A1	SIAH1	TXNIP	NDUFA3
MYC	SLC2A3	SOX4	ZBTB7A	PPP1R15B
NACC1	SLC30A7	STAT3		PTEN
NACC2	SLC39A7	TP53		SOD2
NFAT5	SNAP91	WEE1		SQSTM1
NR2C2	STX4			TXNIP
PIK3R3	TBC1D10C			VEGFA
POU2F1	VAMP2			ZNF207
PTEN	VAMP3			
RAN				
RAPGEF1				
SKI				
STAT3				
TNKS2				

Table S2. List of genes associated with the mechanisms
